# Lead Toxicity: Health Hazards, Influence on Food Chain, and Sustainable Remediation Approaches

**DOI:** 10.3390/ijerph17072179

**Published:** 2020-03-25

**Authors:** Amit Kumar, Amit Kumar, Cabral-Pinto M.M.S., Ashish K. Chaturvedi, Aftab A. Shabnam, Gangavarapu Subrahmanyam, Raju Mondal, Dipak Kumar Gupta, Sandeep K. Malyan, Smita S. Kumar, Shakeel A. Khan, Krishna K. Yadav

**Affiliations:** 1 School of Hydrology and Water Resources, Nanjing University of Information Science and Technology, Nanjing 210044, China; amitkdah@nuist.edu.cn; 2 Central Muga Eri Research and Training Institute, Central Silk Board, Jorhat, Assam 785000, India; aftab.csb@gov.in (A.A.S.); subrahmanyamg.csb@gov.in (G.S.); 3 Geobiotec Research Centre, Department of Geosciences, University of Aveiro, 3810-193 Aveiro, Portugal; marinacp@ua.pt; 4 Water Management (Agriculture) Division, Centre for Water Resources Development and Management, Kozhikode, Kerala 673571, India; ashispc@cwrdm.org; 5 Central Sericultural Germplasm Resources Centre (CSGRC), Central Silk Board, Ministry of Textiles, Thally Road, Hosur, Tamil Nadu 635109, India; rmtarapur@gmail.com; 6 ICAR-Central Arid Zone Research Institute Regional Research Station Pali Marwar, Rajasthan 342003, India; deepak.gupta@icar.gov.in; 7 Institute of Soil, Water and Environmental Sciences, Agricultural Research Organization (ARO), The Volcani Center, Rishon LeZion 7505101, Israel; sanm@volcani.agri.gov.il; 8 Department of Environment science, J.C. Bose University of Science & Technology, YMCA, NH-2, Sector-6, Mathura Road, Faridabad, Haryana 121006, India; smita@jcboseust.ac.in; 9 Centre for Environment Science and Climate Resilient Agriculture, ICAR-Indian Agricultural Research Institute, New Delhi 110012, India; shakeel.khan@icar.gov.in; 10 Institute of Environment and Development Studies, Bundelkhand University, Kanpur Road, Jhansi 284128, India; envirokrishna@gmail.com

**Keywords:** lead toxicity, lead contamination, health hazards, remediation

## Abstract

Lead (Pb) toxicity has been a subject of interest for environmental scientists due to its toxic effect on plants, animals, and humans. An increase in several Pb related industrial activities and use of Pb containing products such as agrochemicals, oil and paint, mining, etc. can lead to Pb contamination in the environment and thereby, can enter the food chain. Being one of the most toxic heavy metals, Pb ingestion via the food chain has proven to be a potential health hazard for plants and humans. The current review aims to summarize the research updates on Pb toxicity and its effects on plants, soil, and human health. Relevant literature from the past 20 years encompassing comprehensive details on Pb toxicity has been considered with key issues such as i) Pb bioavailability in soil, ii) Pb biomagnification, and iii) Pb- remediation, which has been addressed in detail through physical, chemical, and biological lenses. In the review, among different Pb-remediation approaches, we have highlighted certain advanced approaches such as microbial assisted phytoremediation which could possibly minimize the Pb load from the resources in a sustainable manner and would be a viable option to ensure a safe food production system.

## 1. Introduction

Lead (Pb) is a highly noxious, non-disintegrative heavy metal with a bluish-gray color, an atomic number of 82, molecular weight 207.2, density 11.34 g/cm^3^, and a melting point of 621.43 °F. It can be easily shaped, molded, and used to form alloys through mixing with other metals. It can exist in both organic as well as inorganic form. The inorganic Pb dominantly occurs in dust, soil, old paint, and other different user products, while organic Pb (Tetra-ethyl Pb) is predominantly found in leaded gasoline. Both of these forms of Pb are toxic, however organic Pb-complexes are excessively toxic to biological systems compared to inorganic Pb [1]. Pb is the second most toxic metal after Arsenic (As), comprises 0.002% of Earth’s crust [2,3], and its natural level remains to be below 50 mg kg^−1^ [4]. Although earlier literature did not focus on the biological importance of Pb, recent findings suggest that traces of Pb (~29 ng/g diet) is important for enzyme activities and cellular systems, especially during cell development, hematopoiesis, and reproduction [5].

In general, Pb salts/oxides through atmospheric dust, automobile exhaust, paint, polluted food, and water are the key pathways for human exposure. The food canning industry is also an important source of Pb intake due to its leaching ability into canned foods. Currently, humans are exposed to Pb through dust particles from soil transmitted into homes and/or drinking water. Lead is considered carcinogenic (Group 2B) to humans [6]. Humans are impacted by Pb primarily through ingestion as 20–70% of ingested Pb is absorbed by the human body. Children have a high absorption capacity of Pb [7,8].

Enhanced Pb concentration in blood affects behavior, cognitive performance, postnatal growth, delays puberty, and reduces hearing capacity in infants and children. In adults, Pb causes cardiovascular, central nervous system, kidney, and fertility problems. During pregnancy, Pb can also hamper fetal growth in the early stage [1,3]. The Commission Regulation E.C., No 1881/2006, documented the Pb concentration (0.3 mg kg^−1^) thresholds for different agriculture products such as leafy vegetables and fresh aromatic herbs [9]. Pb sources, their inclusion in soil, Pb bioavilability to plants, soil role for Pb transfer to plants, plant toxicity and accumulation mechanism, Pb effect on plants and humans, and different remediation technologies are basically covered in the present review. The main objective of this review is to summarise the research updates on Pb toxicity, bioavailability, and its imposed toxic effects on plants and on human health, including recently tested/recommended remediation options.

## 2. Methodological Approach for Selecting and Reviewing the Literature in a Meaningful Way for Targeting Specific Objectives

### 2.1. Collection, Compilation, and Identification of Relevant Literature for the Study

The criteria for selection of recent literature for targeting up-to-date information on the topic was done through search string/keywords such as “Lead”, “sources of Lead”, “Lead toxicity”, “bioaccumulation of Pb in food and human”, “toxic forms of Pb”, “Pb tolerance in human and plants”, “health effect of Pb toxicity”, and “Pb remediation”. The extensive search of existing literature on the specific keywords was performed to collect the data from Scopus, Science Direct and Google scholar, MDPI, and other academic university websites. Three important criteria were considered for addressing relevant updated information (i) peer-reviewed, (ii) highly cited (i–10), and (iii) articles appearing in journals with a minimum impact factor (>1.0, *Thomson Reuters*) (iv) few articles except the above listed criteria based on recent/specific information was also included. The selection criteria/rules were adopted and modified from Sandin and Peters [10].

### 2.2. Extraction of Data and Data Representation

All the available relevant literature was studied carefully based on the key objectives of the present review. Omission of work was based on literature that was published before 2000, was without quantitative results, non-English, and/or was general/duplicate/similar in nature, which did not fit the questions of this review. Later, the results from all representative literature published from the year 2000 onwards were extracted and represented in tabular form.

## 3. Sources of Pb Contamination in Soil, Crops, and Water Resources

Pb contamination in air, soil, and water resources has been associated with natural causes, such as geochemical weathering, sea spray emissions, volcanic activity, and remobilization of sediment, soil, and water from mining areas [11,12,13]. Table 1 represents the various sources of lead contamination in agricultural soils, crops, and water in different countries/regions of the world. It is evidenced in Table 1 that the anthropogenic products and processes (such as industrial, oil-processing activities, agrochemicals, paint, smelting, mining, refining, informal recycling of lead, cosmetics, peeling window and door frames, jewelry, toys, ceramics, pottery, plumbing materials and alloys, water from old pipes, vinyl mini-blinds, stained glass, lead-glazed dishes, firearms with lead bullets, batteries, radiators for cars and trucks, and some colors of ink) are considered to be major sources of Pb contamination in the environment [14,15,16,17,18,19,20,21].

Pb is available in soil/sediments as a free metal ion, is associated with inorganic molecules (e.g., HCO_3_^−^, CO_3_^2−^, SO_4_^2−^, and Cl^−^), and can also exist as organic ligands (e.g., amino acid, fulvic acid, and humic acid). Pb can also be adsorbed onto particle surfaces such as biological material, oxides of iron, clay particles, and organic matter [22,23]. In general, a higher concentration of anthropogenic Pb accumulates on the soil surface and can decrease with depth [24]. Pb has a high affinity with organic and colloidal materials and, thereby, is readily available for plant uptake [25].

## 4. Pb Bioavailability in Soil and Its Influencing Factors

Lead bioavailability in soil is strongly controlled by its species, especially free-Pb ions concentration [22,34]. Plants absorb lead in dissolved form via the soil solution [25]. Moreover, the concentration of the free lead ion in soils depends on its physical process (e.g., adsorption/desorption) [23].

The behavior of lead species (bioavailability, mobility, and solubility) in soil is controlled by complex interactions of different biogeochemical factors [25]. These factors are redox conditions [35], pH [23,36], cation-exchange capacity [23], soil mineralogy, biological and microbial conditions [2], lead quantity [26,37,38], inorganic and organic legend concentration [22,34,39], competing cation concentration [40,41], and the type of plant species involved [37]. The behavior (uptake rate) of lead species in soil and plants is influenced by either biogeochemical factor independently or in combination with geochemical factors. The effects of some factors on Pb bioavailability are summarized below:

### 4.1. Soil pH

Soil pH is the most important factor that controls Pb availability to plants. Soil pH dictates Pb availability in soil as a negative correlation between Pb solubility and soil pH is noticed [42]. In acidic soil (pH < 7), Pb exists as aqueous Pb(H_2_O_6_)^+2^, while in alkaline soil (pH > 7), Pb forms aqueous complexes with OH^−^ (hydroxyl ions). Specific adsorption of Pb is directly proportional to soil pH [16,43]. At a low soil pH (3–5), adsorption is the dominant process, whereas at a high pH (6–7), precipitation is the dominant process [16,44].

### 4.2. Soil Redox Potential

Redox potential controls Pb dynamics in soil. The solubility of Pb is inversely proportional to soil redox potential (i.e., Pb solubility increases along with a decrease in soil redox potential). Generally, heavy metals dissolve easily in waterlogged soils. Pb was dissolved by acetic acid in highly impeded drainage soil (1.9 μg g^−1^) as compared to freely drained soils (0.1 μg g^−1^) in a region of slate bedrock [16].

### 4.3. Soil Texture

Soil texture significantly affects Pb solubility. In Clay soils, heavy metal ions are adsorbed through ion exchange and specific adsorption mechanisms [45]. Pb adsorption also varies between types of clay minerals [16]. For example, the affinity between iolite and Pb is ~32 times higher than montmorillonite [46]. Mao et al. [47] observed low Pb adsorption on montmorillonite due to competition between Ca and Pb for cation exchange sites on clay.

### 4.4. Soil Minerals

Soil minerals such as Mn and Fe affect Pb solubility in soils. Mn oxides have a high affinity towards Pb, thus they significantly decrease Pb uptake by plants grown in Pb contaminated soil [2,16,48,49,50]. O’Reilly and Hochella [50] emphasized that microbial activity is responsible for Pb mobilization from oxides and carbonate. Tao et al. [51] reported that earthworms could enhance Pb availability to plants.

### 4.5. Nutrients, Organic Carbon, and O_2_

These are the essential factors for microbial growth and metabolism and are directly involved in the degradation of contaminants. Some of the bioactive nutritional elements include carbon (e.g., backbone of all organic compounds), nitrogen (e.g., cellular protein and cell wall component synthesis), phosphorus (e.g., cell membrane, ATP, and nucleic acid), sulfur (e.g., amino acid synthesis), calcium (signaling transport), and magnesium (e.g., enzymatic activities functioning) [52,53] etc. Zhao et al. [54] concluded that soil physical properties such as permeability and fracturing could also affect Pb dynamics in soils. Li et al. [48] elucidated the effect of soil organic matter (OM) on Pb solubility through the formation of complexes during metals’ interaction. Kögel-Knabner et al. [49] emphasized that soil OM drives a sizeable amount of Pb concentration by the formation of organo-Pb complexes.

The ion exchange capacity (particularly CEC), pH, ion redox potential, microbial community, texture, mineralogy, and organic matter of soils are the key regulating factors that affect Pb dynamics (e.g., adsorption, solubility, and mobility) in soil and bioavailability to plants.

## 5. Lead Bioavailability/Bioaccessibility in Animals and Humans

Lead toxicity is an important environmental health hazard and its effects on the human body are devastating. Total Pb in a human body is subject to environment, age, and occupation. It is estimated that a person weighing 70 kg will have an average of 120 mg of Pb, with 0.2 mg/L in the blood, 5–50 in their bones (in mg/kg), and 0.2–3 in tissues [55]. The Center for Disease Control and Prevention (USA) has set the standard elevated blood Pb levels for both adults and children (10 μg/dL and5 μg/dL, respectively) [56].

Bioavailability (BA) is an ingested fraction that crosses the gastrointestinal epithelium and is distributed into internal tissues and organs [57]. Bioavailability of Pb was established through in-vivo models such as in mice (*Mus*), monkeys (*Cercopithecidae*), rabbits (*Oryctolagus cuniculus*), rats (*Rattus*), and swine (*Sus scrofa*). However, extrapolation of the in-vivo models into human has not provided a realistic effect due to their physiological differences and species diversity. In-vivo experiments are much simpler than epidemiological studies because they are cheaper, faster, highly reproducible, and do not involve ethical issues. However, critical parameters (e.g., exposure levels, conditions, and absorbed Pb concentration) need to be considered while performing *in-vivo* specimen evaluation. The following key factors are to be considered for decision making in public health issues using in-vivo models: (a) specific features and limitations of the model; (b) targeting the human population in the design of animal studies at developmental stage; (c) the use of acceptable environmental doses, and (d) Pb speciation. In-vitro studies such as Relative Bioavailability Leaching Procedure (RBALP), Unified Bio-accessibility Research Group Europe Method (UBM), Solubility Bio-accessibility Research Consortium assay (SBRC), Physiologically Based Extraction Test (PBET), In Vitro Gastrointestinal (IVG) Method, and the In Vitro Digestion Model (RIVM) can be used to measure Pb bioaccessibility [58]. Pb relative bioavailability (RBA) refers to the comparative bioavailability of different Pb forms that are available in source substance [58]. For estimating the relative bioavailability of Pb, a reference material such as Pb acetate can be used. Lead RBA in soil can be measured by either blood or tissues (kidney, liver, and femur) [58,59]. Deshommes et al. [60] conducted an in-vivo experiment on Pb particles (especially particulate Pb forms including those in paint and dust and those in drinking water supply systems) and stated that the relative bio-accessibility leaching procedure (RBLP) offers the highest degree of validation and simplicity in animal models.

Literature suggests that due to unavailability of data and the existing model (e.g., animal model), we could not predict/estimate human risk assessment and human absorption of Pb particles, particularly for childhood exposure assessment, e.g., neuro-behavioral and neuro-developmental deficiencies, and the effects on growth, hearing, and blood pressure.

## 6. Lead Transportation, Toxicity, and Bioaccumulation Through Food Chain Contamination

Lead is one of the most toxic and frequently encountered heavy metals in the environment [34]. Different quantitative indices are currently being used to estimate Pb toxicity at trophic levels in the food chain (Table 2). Once Pb reaches the soil by any source and penetrates into the plant root system, it may accumulate there or may be translocated to aerial plant parts (APP). Pb mostly accumulates (≥95%) in the roots of plant species and only a small fraction is translocated to APP. Some of the studied plants species with respect to Pb transportation, toxicity, and bioaccumulation are *Allium sativum* [61], *Avicennia marina* [62], *Pisumsativum*, *Phaseolus vulgaris* and *Vicia faba* [34,63,64], *Lathyrus sativus* [65], *Nicotiana tabacum* [66], *Sedum alfredii* [67], *V. unguiculata* [68], and *Zea mays* [69].

Generally, plants uptake metal ions from soils through their roots [17,18,20]. Pb from the soil solution is adsorbed (unevenly) through roots and is bound with the uronic acid/polysaccharide of rhizoderm in many plant species such as *Brassica juncea* [70], *Festuca rubra* [71], *Funaria hygrometrica* [72,73], *Lactuca sativa* [74], and *Vigna unguiculata* [68]. This adsorbed Pb passively enters in roots and is transported through xylem. A concentration gradient was observed near the root apex, except for root cap, where cells are young and have thin cell walls with the lowest rhizodermic pH, which enhances Pb solubility in soil solution.

After entering into the roots, Pb moves by apoplast through water stream until it reaches the endodermis region. The endoderm functions as a physical barrier to Pb translocation as water stream is blocked by casparian strip and, thus, Pb enters into the symplastic movement. The low Pb transportation from root to APP has been reported due to immobilization by negatively charged pectins within the root cell wall [2,84]. Insoluble Pb salts precipitate in intercellular spaces of root cells [70,84]. Similarly, Pb accumulation in plasma membranes of root cells [61,84] or sequestration in the vacuoles of rhizodermal and cortical cells of roots is reported [68,84]. The major portion of the absorbed lead is sequestered/excreted from endodermis cells during the plant detoxification process. However, the above reasons are not sufficient to explain the low Pb translocation from root to APP as plant species such as *Brassica pekinensis* and *Pelargonium* potentially translocate Pb to APP, without affecting metabolic functions [85,86]. The lead hyper accumulator plant species can accumulate >1000 ppm [87]. The roots of hyperaccumulator species dissolve metals in soil [86], increase metal uptake and translocation, and make hyperaccumulator species to tolerate higher Pb ions concentrations. Apart from this, various detoxification mechanisms include selective metal uptake, excretion, complexation by specific ligands, and compartmentalization, which are also support for Pb tolerance.

In addition, Pb translocation to APP increases by organic chelators like ethylene diamine tetra acetate (EDTA) and micro-organisms [2,25]. Liu et al. [88] observed higher translocation to APP with increased soil Pb level in *B. Pekinensis* cultivars. This may be due to the potential of high Pb concentrations to destroy the casparian strip based physical barrier.

Xylem helps in the transportation of metals from plant roots to shoots [89], which is probably supported by transpiration [90]. Arias et al. [2] demonstrated X-ray mapping and found high Pb deposition in xylem and phloem cells on mesquite plants. After penetrating into the central cylinder of the stem, Pb can again be transported via the apoplastic pathway and further translocated to leaf areas through vascular flow [73]. In xylem, Pb can form complexes with amino/organic acids [87]. However, inorganic Pb can also be transferred. Translocation factor (i.e., lead in aerial parts/leading roots) can be implemented to know the degree of Pb translocation [86,88]. After implementing this factor, low numeric values will indicate that lead has been sequestered in the roots system [88].

The molecular mechanism of Pb entrance in roots is not clear yet. It is believed that several pathways can be used by Pb for the same purpose, especially ionic channels. However, Pb uptake is a non-selective phenomenon and is independent of the H^+^/ATPase pump [91]. Lead absorption is inhibited by calcium [92] as Pb competes with Ca for calcium channels. Ca^2+^-permeable channels are important gateways for Pb to penetrate into the root system [91,93]. The transgenic plant studies reveal that Pb can also penetrate into roots through other alternative non-selective pathways, e.g., cyclic nucleotide-gated ion channels and low-affinity cation transporters [94]. Comprehensive details for the average lead content in different food crop plants are summarized in Table 3. It is noted that higher concentrations of Pb are associated with fruit crops (Table 3).

Accidental soil ingestion is a major Pb exposure pathway for humans inhabited in a Pb polluted area [9,139]. However, the intake of Pb contaminated plants has been an important exposure to humans and animals [9,139,140]. Edible/wild plants cultivated/grown in the vicinity of phosphate industries can be Pb bio-indicators of toxic metals [9]. Inhabitants and workers of these industries/provinces may be exposed to Pb contamination. The Pb exposures and blood concentration to these closely inhabited/living populations is subject to the season as well as industrial activity. The children’s blood lead levels (BLLs) were observed to be higher during the summer and early fall [141]. The BLLs are highly significant, are evident in multiple locations, periods, and ages, and are population-specific [142,143]. Higher levels were observed (10–60%) in warm-weather and levels increased in 2-year-old children, more so than 1 or over 4-year-olds [142,143]. Zahran et al. [143] emphasized that lead seasonality must be considered for Pb risk analysis. One health concept was proposed to take care of animal, human, and environmental health all together [144,145].

## 7. Mechanistic Understanding of Pb Toxicity and Tolerance in Plants and Humans

Lead causes a broad range (physiological, morphological, and biochemical) of toxic effects on living organism. In plants, Pb toxicity is characterized with impaired chlorophyll (Chl a) production, cell division, elongation of root, lamellar organization in the chloroplast, plant growth, seed germination, seedling development, and transpiration [67,87]. However, the magnitude of the effects varies and/or depends on Pb levels, exposure time, plant stress intensity, and plant developmental stage. Plants have internal detoxification mechanisms to deal with Pb toxicity, i.e., complexation by specific ligands, selective metal uptake, excretion, and compartmentalization [18,21,61,87].

Lead induced oxidative stress is reported to produce reactive oxygen species (ROS) in plants [146,147]. These ROS synthesized as a result of oxidative stress in plants can cause deleterious effects such as lipid peroxidation, disrupted cell membrane, DNA and protein damage, inhibition of photosynthesis, and inhibition of ATP production [148]. To overcome the adverse effects of ROS, plants produce a variety of antioxidative enzymes. Lead imposed changes in antioxidative enzyme production of various food crops have been well established (Table 4). The activity of antioxidative enzymes, such as superoxide dismutase, peroxidase, and ascorbate peroxidase, were positively correlated with Pb content, while Catalase, Glutathione reductase, and Glutathione peroxidise were decreased in both leaf and root tissues (Table 4).

Lead poisoning cases in humans are mostly the result of oral ingestion and absorption via the gut [149]. Pb absorption from the gastrointestinal tract is subject to physical characteristics (such as age, pregnancy, fasting, and Fe and Ca status) and the physico-chemical nature of the material ingested (e.g., size of particles, solubility, mineralogy, and Pb species) [150]. The Pb absorbed in the intestine is further carried to soft tissue, e.g., in the liver, kidneys, and bone tissue, where it accumulates over time [149]. The main transport processfor Pb to different body tissues from the intestine is via red blood cells, where binding takes place between Pb and haemoglobin (HB). Nearly 99% of the Pb in blood is observed in erythrocytes, with approximately 1% in both serum and plasma. Distribution of Pb in the organs (the lungs, spleen, brain, aorta, renal cortex, bones, and teeth) relies greatly on Pb concentration in plasma rather than on the whole blood. The half-life of Pb in blood is estimated to be 35 days, whereas the half-life of Pb in soft tissue is estimated to be 40 days. Pb can be resident in bone for up to 30 years and concentrations of Pb in teeth and bone grow in proportion to age [149]. The Pb biological half-time is believed to be significantly greater in children than in adults. Lead creates chemical bonds with thiol groups of proteins and Pb toxicity is believed to inhibit enzymes and subsequently interfere with homeostasis of Mg, Ca, and Zn. Lead-induced oxidative stress is caused due to Pb poisoning as it disrupts the pro-oxidant/antioxidant cell defence system. Antioxidant nutrients, such as vitamins E, C, B_6_, and B-carotene, and also Zn and Se, are believed to combat Pb-induced oxidative stress [151].

High levels of Pb absorption are found in children rather than in adults. It is approximated that adults may absorb 3–10% of an oral dose of water-soluble Pb, whereas for children, it may be as high as 40–50%. Higher Pb concentrations are found in the blood of children who are Fe- or Ca-deficient than those with replete Fe or Ca. Pb absorption may raise during the pregnancy period and over 95% of Pb deposits in skeletal bones as insoluble phosphate [149]. According to autopsy studies, cortical bone and teeth together account for 90–95% of the body’s Pb burden. The total Pb body burden in the skeleton is 80–95% in adults and about 73% in children [149]. Mothers may transfer Pb to the foetus and also to infants during the period of breastfeeding [152]. Pb toxicity principally targets the human central nervous system and children’s ingestion of large amounts of Pb from the environment, particularly when anaemic, is linked to lower intelligence and impaired motor function [149].

The Joint FAO/WHO Expert Committee on Food Additives (JECFA) made an estimation of tolerable weekly intake based on dose-response analyses and concluded that the provisional tolerable weekly intake (PTWI) is linked to a reduction in children’s IQ of at least 3 points and systolic blood pressure of approximately 3 mmHg (0.4 kPA) higher in adults [149]. When observed in terms of a shift in IQ distribution or blood pressure in a population, these changes assume greater importance. The JECFA’s conclusion, therefore, was that the PTWI is no longer adequately protective of health and they withdrew it. The lack of an indication of a threshold level for key effects of Pb based on the dose-response analysis led the JECFA to conclude that a new PTWI considered as health-protective could not be established. The JECFA reiterated that foetuses, infants, and children are the subgroups that have the highest sensitivity to Pb [150,153] due to the neuro-developmental effects. The European Commission [154] has set guidelines for maximum permissible levels of Pb in some foodstuffs (Table 5). Interventions such as eliminating leaded petrol, banning the use of Pb in wine bottles, and the discontinuation of soldered cans are seen as an important factor in successful reduction of Pb in food. In children, Pb toxicity symptoms are loss of appetite, anemia, behavioral changes, delayed mental growth and learning, fatigue, headaches, hyperactivity, insomnia, metallic taste, reduced nerve conduction, weight loss, and possibly neuron disorders [155]. The behavior changes are irreversible and untreatable as the cerebrum of *Homo sapiens* has little capability for reparation. A daily Pb intake of up to 7 μg/kg body weight or 490 μg of Pb for an adult was accepted by WHO, FAO. However, no such guideline is given for infants and children, who are relatively more sensitive to low Pb levels [156].

In broilers that have high Pb acetate (200 mg/kg) exposure in their diet, these show anorexia, greenish diarrhea, leg paresis, weight loss, wing droop, and lethargy symptoms including gross change in kidney, spleen, and liver function, gizzard lining, hemorrhages on muscles [157], etc. Gao et al. [157] concluded that Pb could alter the expression of selenoprotein related genes in the cartilage tissue of broilers. Rahman and Joshi [158] revealed that Pb acetate (i.e., 250–400) in drinking water could lead to reduced feed intake and growth indices in broilers due to higher oxidative stress. Pb-induced oxidative stress can also reduce antioxidant activities such as catalase, glutathione superoxide dismutase [159], etc. and erythrocytes burst due to lipid peroxidation in erythrocytes membranes and may cause hemolytic anemia [160]. Pb could also respond to change in the activities and expression of antioxidant enzyme–related genes [161,162]. Most animal experiments confirm that Pb transportation in the body occurs through blood circulation and accumulates in soft tissues, bones, and other pivotal organs [163]. Bones are a major sink of Pb (~90%) and mostly replace calcium, thus decreasing in bone mineral density (BMD) due to Pb exposure [164].

## 8. Human Health Effects Due to the Consumption of Pb Contaminated Foodstuffs

Lead enters into the body through pathways like inhalation of wind-blown Pb-laden dust, ingestion of Pb contaminated soils, oral intake of Pb contaminated water, and food grown in Pb-contaminated areas. Pb accumulation in livestock tissues may also pose a major risk to human health through livestock meat consumption [173,174]. After absorption, Pb is distributed in the body through red blood cells (RBC). Pb is mostly bound to hemoglobin rather than RBC membrane after entering the cell [175]. The hematopoietic is a sensitive system for critical Pb toxicity and may lead to anemia [160]. Histopathological observations confirmed that Pb ions are transported to the liver, where they can induce chronic damage to the liver. Pb toxicity also increases blood enzyme levels and reduces protein synthesis [176,177,178]. Pb imposes toxic effects on kidneys through structural damage and changes in the excretory function [176,177,179]. The other organ and tissue systems affected due to lead toxicity are the nervous, cardiovascular, and reproductive systems [160,175,180]. Pb toxicity imposes mineralizing of bones and teeth, which is a major body burden [3]. The International Agency for Research on Cancer (IARC) stated that inorganic Pb is probably carcinogenic to humans (Group 2A) based on limited evidence in humans and sufficient evidence in animals [181]. Generalized clinical symptoms of Pb poisoning in humans are comprehensively summarized in Table 6.

## 9. Pb Remediation approaches

Innovative and site-specific Pb remediation technologies for efficient clean-up of contaminated sites are prerequisites for a healthy life and safe food production. There are different (physical, chemical, and biological) processes developed to reduce total Pb concentration and Pb bioavailability to mitigate Pb accumulation in the food chain [182,183].

### 9.1. Physical Approaches

#### 9.1.1. Replacement of the Medium (Soil/Water)

In this method, the complete or partial replacement of the contaminated resources (soil/water) is done based on the magnitude of the contamination [183]. This method of remediation is very useful at a small scale at the local level. The biggest challenge for this method is the safe disposal of the contaminated soil/water in a cost-effective manner.

#### 9.1.2. Vitrification

This method can be applied through both in-situ and ex-situ remediation mechanisms. In vitrification methods, soil is melted with the help of a high-temperature process and Pb sequestration achieved in solidified vitreous mass [183,184]. Vitrification can be used long-term and effective low volume can be obtained for reuse [183]. This is a costly method and may not be suitable for applications in large areas. Dellisanti et al. [185] carried out the vitrification of Pb-rich ceramic waste. Wang et al. [186] treated fly ash from a municipal solid waste incinerator to radiated heavy metals including Pb. Navarro et al. [187] applied vitrification for remediating the hazardous mine wastes from old mercury and Ag-Pb mines in Spain.

#### 9.1.3. Electrokinetic Remediation

Electrokinetic remediation is achieved by applying current in the field. This process involves techniques such as electrophoresis, electric seepage/electro-migration, electro-osmosis, and electrolysis [188]. Kim et al. [189] has shown that contaminated rice soil could be cleaned using an electrokinetic technique, which reduces Pb contamination by 19.4% in 4 weeks. Jeon et al. [190] remediated a soil contaminated with Pb in a paddy rice crop using EDTA as an electrolyte. The electrokinetic remediation technique generates almost nil waste. Electrokinetic remediation is applicable for saturated soils with low groundwater flow, requires short repair time and low energy, and provides a complete repair [183]. The heterogeneity of soil and treatment depths are the two important limitations of this method.

### 9.2. Chemical Approaches

Various chemical amendments are widely used for immobilization of lead in soil and ground water at the field scale (Table 7).

#### 9.2.1. Chemical stabilization

This method is used to decrease the mobility, bioavailability, and bio-accessibility of heavy metals in soil. The immobilizing agents, i.e., biochar (Wheat, Rice, Miscanthus straw biochar, Sugarcane bagasse biochar, Holm oak chips biochar), clay minerals (Sepiolite with limestone, Palygorskite, and Bentonite), liming materials (Oyster shells and eggshells), metal oxides (Mn oxides and Ferric oxyhydroxide powder with limestone), organic composts (Biosolid), and phosphate compounds (Phosphate rock, Calcium magnesium Phosphate, and Single superphosphate) were previously used in the chemical stabilization process [183], details of which are given in Table 7. Chemical stabilization is a simple, quick, relatively cost-effective chemical approach by which Pb can be immobilized by adsorption, chemical precipitation, ion exchange, and surface complexation mechanisms to limit Pb transport and bioavailability. However, in this process, Pb remains in the soil and hence, long-term immobility is recommended.

#### 9.2.2. Solidification/Stabilization

Solidification/stabilization (waste fixation) is relatively low cost, low risk, easily implemented, and highly resistant to biodegradation with abroad engineering applicability [183]. Soil solidification refers to the encapsulation of waste materials in a monolithic solid with high structural integrity [183,191]. Soil stabilization is achieved by stabilization of soil contaminants through chemical interaction between Pb and binding reagents [192]. Wang et al. [193] and Antemir et al. [194] demonstrated the potential cement-based binders in remediating heavy metals including Pb in England. Navarro-Blasco et al. [195] assessed the Pb adsorption capacities of calcium aluminate cement. Voglar and Lestan [196] used calcium aluminate cement and sulfate resistant Portland cement as binders for Pb immobilization in Slovenian soil. Wang et al. [197] assessed Portland cement, ground granulated blast furnace slag, pulverized fuel ash, MgO, and zeolite for on-site soil solidification/stabilization of Pb in UK soil.

#### 9.2.3. Soil Washing

The soil washing process is achieved by Pb leaching from soil matrix using reagents/extractants such as chelating agents, inorganic acids, organic acids, surfactants, and water. The soil mixing with respective reagents/extractants is done where extractants transfer Pb from soil to the liquid phase through chelation or desorption, chemical dissolution, and ion exchange mechanisms [198]. Soil washing is a rapid, permanent, effective chemical method for Pb remediation with long term liability [199]. Hu et al. [200] achieved removal of 73% Pb using EDTA as a chelating agent. Wang et al. [201] used iminodisuccinic acid, glutamate-N, N-diacetic acid, glucomonocarbonic acid, and polyaspartic acid to extract 53% and 55% Pb from Pb-Zn contaminated soil. However, these technologies have certain hurdles for their practical utility due to change in soil properties, loss of nutrients, adverse effect of washing chemicals, generation of wastewater, and cost of chemicals and their negative impact on the environment.

### 9.3. Biological Approaches

Biological methods for Pb remediation are the most eco-friendly alternatives to remediate Pb from the contaminated resources. Biological remediation can be referred as direct utilization of any natural/genetically engineered living organism and their product for Pb detoxification to restore soil function and quality.

#### 9.3.1. Phytoremediation

Phytoremediation is an environmentally-friendly, attractive, aesthetically pleasing, noninvasive, energy-efficient, and cost-effective technology that can remediate Pb in low to moderate contaminated soil. It includes phytostabilization and phytoextraction. Phytostabilization decreases the mobility/bioavailability of Pb through adsorption by roots, chemical precipitation, and complexation in the root zone. Phytostabilization is only effective up to the root depth of plants. Cheng et al. [217] observed the Pb phytoremediation potential of *Miscanthus floridulus.* Yang et al. [218] ascertained the phyto-extraction potential of a co-planting system of *Pteris vittata* L. and the *Ricinus communis* L. in Pb contaminated soil and observed an increased yield of *P. vittata* after Pb uptake. Metal hyper accumulater plant species such as *Eichhornia crassipes*, *Lemna* sp., *and Pistia stratiotes* have been widely used to remediate Pb from diversified environments (Table 8).

#### 9.3.2. Microbial Remediation

Microbial remediation refers to decreasing the availability of Pb in the environment using indigenous/exotic microbes. Bacterial species such as *Alcaligenes* sp., *Bacillus firmus*, *Bacillus licheniformis*, *Enterobacter cloacae*, *Escherichia coli*, *Micrococcus luteus*, *Pseudomonas fluorescens*, and *Salmonella typhi* show adsorption potential of Pb from the contaminated resources [219,220,221,222,223]. Wang et al. [224] concluded that bacterial strain B38 (mutant of *Bacillus subtilis*) has immense potential to remediate heavy metals including Pb in China. Zeng et al. [225] observed that *Aspergillus niger* strain SY1 effectively removed Pb (99.5%) from contaminated sediment through bioleaching. The fungal biomass of *Lepiotahystrix*, *Aspergillus niger*, *Aspergillus terreus*, and *Trichoderma longibrachiatum* are reported as potential bio-sorbents [223,226,227]. The algal species i.e., *Palmaria palmate*, *Spirulina maxima*, *Spirogyra hyaline*, *Cystoseira barbata*, *Cladophora* sp., *Chara aculeolata*, *Nitella opaca*, and *Ulva lactuca* are were identified to be efficient bio-sorbents [223,228,229]. Microbial remediation is considered to be a natural, safe, and effective eco-friendly technology with low energy and low operation cost inputs [183]. Most importantly, microbial remediation does not impose any environmental and health hazards. The process depends on the environmental condition and inputs such as nutrients, oxygen, and other amendments to stimulate microbial activity for Pb remediation [183].

#### 9.3.3. Microbial Assisted Phytoremediation

Many approaches including molecular fingerprinting techniques *viz.* length heterogeneity analysis by PCR (LH-PCR), terminal restriction fragment length polymorphism (T-RFLP), denaturing gradient gel electrophoresis (DGGE), single strand conformation polymorphism (SSCP), ribosomal intergenic spacer analysis (RISA), cloning, and In Situ Hybridization (ISH/FISH) were used to identify the potent microbial community involved in phytoremediation [230,231,232,233,234]. This approach is based on the rhizosphere associated microbes such as *Bacillus*, *Beijerinckia*, *Burkholderia*, *Enterobacter*, *Erwinia*, *Flavobacterium*, *Gluconacetobacter*, *Klebsiella*, *Pseudomonas*, *and Serratia* [235,236,237]. Babu et al. [235] inoculated soil with rhizospheric bacteria *Pinus sylvestris* and found significant increases in biomass, chlorophyll content, nodule number, and Pb accumulation in *Alnus firma* seedlings.

### 9.4. Biotechnological and Genetic Approaches

Genomics, metagenomics, metabolomics, proteomics, transcriptomics, nanoparticles, and isotope probing are modern technologies to understand Pb phytoremediation [234,235,257]. The biotechnology and genetic approaches to remediate Pb from the contaminated resources have great potential and have been proved in some plants [19]. Hattab et al. [258] observed a significant increase in ROS and cellular oxidative stress in *Medicago sativa* through influencing the expression of CuZn-SOD, GSH synthase (GS), and GPX against Pb stress. Fan et al. [259] observed an unknown protein, product of PSE1 (Pb-sensitive1) gene with NC domain, which is localized in cytoplasm and has potential for Pb tolerance in *A. thaliana*. Jiang et al. [260] studied the role of PDR12 knockout *Arabidopsis* and under Pb stress conditions and concluded that PDR12 is responsible for the activation of a Pb exclusion mechanism. ABC transporter of the mitochondria 3 (ATM3) [260,261,262], acyl-CoA-binding protein [263], and leucine-rich repeat2 (LRR2) and ethylene-insensitive 2 (EIN2) [264] are also important to regulate Pb transportation to the exterior of the cell [19,265]. A cytosol-localized malate dehydrogenase (CMDH4) protein functions as regulation of Pb tolerance mechanisms [19,266]. Pb is easily affected by GSH reductase in the plant cell [267]. Pb-mediated increased expression phytochelatins were also observed in *Salvinia minima* [268]. *M. sativa* plants showed 23-fold increased expression of PCS gene in the presence of Pb [258]. Furthermore, GMO plants develop efficient metabolic processes and over express genes/enzymes that are capable of bioremediation specific pollutant. Different omic-approaches help to explore different potential solutions targeting precise pollutants. For utilizing the omic-approaches below, certain research should be covered:

(a) Identification of candidate genes for effective and efficient removal of Pb contaminants.
(b) Diversity and phylogenetic studies of gene and protein sequences which control Pb bioremediation.
(c) Development of Genetically modified organism (GMO) plants through transgenesis.

**Figure 1:**
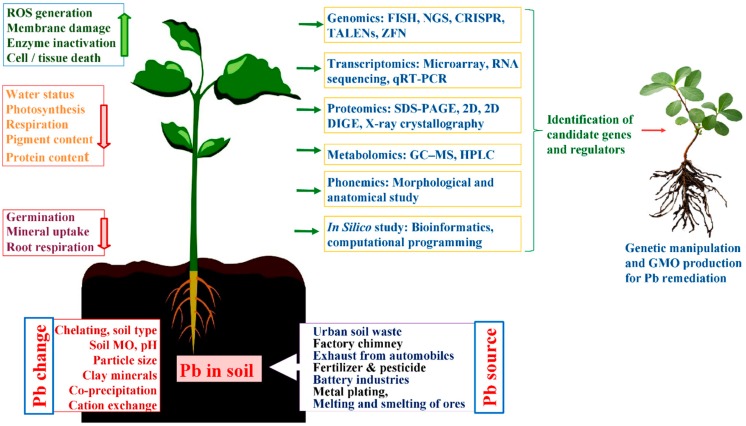
Biotechnological and Genetic Approaches for the development of efficient remediation systems.

GMO plants are capable of remediating various waste effluents and polluted lands and could be advantageous for bioremediation practical applications. Moreover, information on the fundamental omic-approaches concerned in bioremediation can also contribute towards the development of efficient bioremediation systems. Besides that, analysis of comparative genomic and proteomic study, their functional variations, as well as evolutionary relationships existing between them can contribute towards designing new efficient bioremediation systems. Systems biology information like molecular pathways, gene ontogeny analysis, co-expression, and protein-protein interactions can influence the Pb bioremediation processes. Therefore, with the help of bioinformatic analyses and modern biotechnological techniques, one can evaluate and justify the need for genetically modified organisms for the development of efficient remediation systems in the near future (Figure 1).

### 9.5. Nano-Technological Approaches

Recent scientific development in nanoscience research opens the way to cost-effective, eco-friendly, and sustainable remediation approaches. A nano technological approach has been successfully used in soil, sediments, solid waste, and a wastewater remediation [18,269] process. Nano-materials are dynamic, efficient, and broadly applicable with economic expediency [18,270]. The characteristic features of nano-materials such as Nanocatalysts, CNTs, graphenes, nano-scale metal oxides, nanomembranes, carbon nanotubes, nanobiological processes and zero-valent iron (FeO), Fe_2_O_3_, Fe_3_O_4_, TiO_2_, SiO_2_, and Al_2_O_3_ are summarized in Table 9. Nanoparticles (1–100 nm) provide very high adaptability for both in-situ and ex-situ remediation approaches [18]. Nanomaterials, nanoadsorbents, and nanosized compounds (quantum dots, nanofilms, nanoparticles, nanotubes, nanowires, and other various colloids) used for Pb remediation are listed in Table 9. Nanoparticles (less than 50 nm) have high potential as Pb adsorbents. Nano-adsorbents, i.e., activated carbon, alginate biopolymer, clay materials, silica, magnetic iron oxide nanoparticles (MNPs), metal oxides, nano-titanates, etc. have been utilized to remove Pb [18,271,272]. The researchers showed that nano-material can enhance the accumulation of metals by improving the cell wall permeability, co-transportation of nanomaterials with heavy metals, and transporter gene regulation [18,273].

## 10. Conclusion and Future Prospects

The source, bioaccumulation, and health hazards of Pb are due to industrial and agricultural activities. Translocation of Pb from soil to a crop system is a complex and species dependent phenomenon. The human consumptive plant species have shown different bioaccumulation, tolerance, and toxicity levels for lead. Based on the tolerance mechanism, different concentrations of Pb accumulate in the food chain and cause different magnitudes of human health hazards. To minimize these Pb based health risks, different remediation options are available for reducing the concentration of heavy metals in soil and the food chain. However, site and source-specific integrated approaches must be practiced to formulate suitable remediation strategies. Biological remediation, such as phytoremediation and PGPR, can be an environmentally friendly and cost-effective strategy for alleviating Pb toxicity in moderately contaminated soils. Eco-feasible technological innovations such as nano-tools and awareness among farmers’ fraternity could possibly boost local economies and livelihoods with certain financial guarantees. Similarly, suggestive measures should be taken to ensure the sustained efficacy of Pb remediation such as the development of promising plants/mechanisms suitable for Pb phytoremediation. Exploitation of molecular approaches is required to manipulate Pb transporters and their cellular targeting to specific cell types. Development of transgenic plants with enhanced plant-microbe interaction is also a viable option to enhance phyto-remediation of Pb.

## Figures and Tables

**Table 1:** Table of Pb contamination in agricultural soils, crops, and water in different countries [18].

Sources	Contaminati-on	Plant Species	Region	References
Wastewater of Shitalakhya river	Soil and vegetables	*Amaranthus lividus*, *Basella alba*, *Cucurbita moschata*, *Spinacia oleracea*, and *Trichosanthes cucumerina*	Bangladesh	[26]
Wastewater treatment plant	Soil, water, and crops	*Eruca sativa*, *Madia sativa*, *Malus sylvestris*, *Triticum æstivum*, *Triticum turgidum*, *Urtica dioica*, and *Vicia faba*	Morocco	[27]
Mine affected area	Soil and vegetable	*Amaranthus dubius*, *Ipomoea aquatic*, *Ipomoea batatas*, *Phaseolus vulgaris*, *Piper nigrum*, *Solanum lycopersicum,* and *Solanum melongena*	China	[28]
Sewage water	Soil and crop	*Oryza sativa*	Iran	[29]
Agricultural/Urbanisation activities	Water and sediments	*Lemna minor*	India	[30]
Urbanization	Soil, water, and vegetables	*Brassica oleracea*, *Momordica charantia*, *Phaseolus vulgaris*, *Raphanus raphanistrum*, *Solanum lycopersicum*, and *Triticum aestivum*	China	[31]
Anthropogenic activities	Soil and vegetables	*Cucurbita maxima*, *Lagenaria siceraria*, *Solanum melongena*, and *Spinacia oleracea*	Pakistan	[32]
Glass industry	Soil and agricultural crops	*Brassica juncea*, *Hordeum vulgare*, and *Triticum aestivum*	India	[33]

**Table 2:** Different indices used to quantify Lead toxicity at trophic levels in the food chain [18].

SN	Factors	Equations	References
1	Trophic transfer factor (TTF)	TTF = Pb conc. in organism tissue/Pb conc. in food	[75]
2	Transfer factor (TF)	TF = Pb conc. in plant tissue/Pb conc. in soil	[76]
3	Metal transfer factor (MTF)	MTF = Pb conc. in plant/Pb conc. in soil	[77]
4	Accumulation factor (AF)	AF = Pb conc. in plant edible part/Pb conc. in soil	[78]
5	Bioaccumulation factor (BAF)	BAF = Pb conc. in organism tissue/Pb conc. in abiotic medium	[79]
6	Bio-concentration factor (BCF)	BCF = (Pb conc. in experimental organism tissues − Pb conc. in the control organism tissues)/Pb conc. in water	[80]
7	Biota-sediments AF (BSAF)	BSAF = Pb conc. in the organism/Pb conc. in sediments	[81]
8	Biomagnification factor (BMF)	BMF = Pb conc. in the organism/Pb conc. in the organism’s diet	[82]
9	Trophic magnification factor (TMF)	TMF is calculated from the slope of logarithmically transformed Pb conc. in organisms plotted against the trophic levels of the organisms in the food web	[83]

**Table 3:** Details for the average lead contents in different crop plants.

Plant Species	Scientific Name	Concentration (mg/kg)	References
**Vegetable crops**			
Coriander	*Coriandrum sativum*	4.5	[95]
Spinach	*Spinacia oleracea*	0.98–9.2	[96,97,98,99]
Coriander	*Coriandrum sativum*	0.4–75.5	[98,100,101,102,103,104,105]
Cabbage	*Brassica oleracea*	0.07–12	[97,104,106,107,108]
Radish leaf	*Raphanus sativus*	0.4	[100]
Amaranthus	*Amaranthus blitum*	23.26	[109]
Parsley	*Petroselimum crispum*	2.31	[97]
Slender amaranth	*Amaranthus viridis*	2.56	[101]
Sugar beet	*Beta vulgaris L*	149.5	[102]
Slender amaranth	*Amaranthus viridis*	5.44	[110]
Tomato	*Solanum lycopersicum*	5.5	[99]
Brinjal	*Solanum melongena*	2.1	[95]
Cucumber	*Cucumis sativus*	1.5	[95]
Brinjal	*Solanum lycopersicum*	2.2	[98]
Raddish	*Raphanus sativus*	0.75	[111]
Eggplant	*Solanum melongena*	4.93	[112]
Brinjal	*Solanum tuberosum*	6.19	[112]
Pumpkin	*Cucurbita maxima*	0.25	[113]
Chilli	*Capsicum annuum*	0.17	[113]
Carrot	*Daucus carota*	0.72–7.8	[95,96,97]
Sugar beet	*Beta vulgaris* L.	26.35	[109]
Potato	*Solanum tuberosum*	0.012–2.58	[106,107]
Cauliflower	*Brassica oleracea*	0.36–6.1	[95,97,104]
**Spices Crops**			
Aniseed	*Pimpinella anisum*	0.26–5.68	[114,115]
Bay leaf	*Cinnamomum tamala*	0.98–3.58	[116,117,118]
Cardamom	*Elettaria cardamomum*	0.583	[115]
Cassia	*Cinnamonum cassia*	4.159	[115]
Curry	*Murraya koenigii*	3.617	[117]
Dill	*Anethum graveolens* L.	0.81	[119]
Fennel	*Foeniculum vulgare*	0.316	[115]
Fenugreek	*Trigonella foenum-graecum* L.	9.38	[114]
Rosemary	*Rosmarinus officinalis*	10.8	[120]
Tulsi	*Ocimum sanctum*	4.59	[116]
**Fruit Crops**			
Mango	*Magnifera indica*	0.642–1.620	[121,122]
Orange	*Citrus sinensis*	26	[123]
Pomegranate	*Punica granatum*	28	
Grapes	*Vitis vinifera*	24	
Lemon	*Citrus limon*	29	
Strawberry	*Fragaria ananassa*	10	
Buckthorn	*Hippophae rhamnoides*	20	
Peaches	*Prunus persica*	11	
Banana	*Musa* sp.	0.003–0.05	[122,124]
Jackfruit	*Artocarpus heterophyllus*	0.017	
Orange	*Citrus sinensis*	0.106	[125]
Trengerine	*Citrus tangernia*	0.097	
Banana	*Musa*	0.118	
Papaw	*Carica papaya*	0.072	
**Cereals and Legumes Crops**			
Pearl millet	*Pennisetum glaucum*	0.12	[126]
Sorghum	*Sorghum bicolor*	0.18	[126]
Wheat	*Triticum aestivum*	0.40	[127]
		0.47	[128]
Barley	*Hordeum vulgare*	0.22	[129]
Quinoa	*Chenopodium quinoa*	0.37	[130]
Maize	*Zea mays*	0.50	[131]
		0.34	[132]
		0.31	[133]
Rice	*Oryza sativa*	0.52	[134]
		0.89	[135]
Black gram	*Vigna mungo*	0.60	[133]
Lentil	*Lens culinaris*	0.55	[133]
Common bean	*Phaseolus vulgaris*	0.12	[136]
Soybean	*Glycine max*	0.08	[137]
Safflower	*Carthamus Tinctorius*	0.80	[138]
Rapeseed	*Brassica napus*	0.51	[138]
Sunflower	*Helianthus annus*	0.57	[131]

**Table 4:** Effects of Pb toxicity on activities of different antioxidant enzymes in different plants [16].

	Enzymes		Pb Exposure Level	Duration	References
	Enhanced	Reduced			
*Sedum alfredii*	SOD	APX	0–200 μM	14	[63]
*Triticum aestivum*	SOD, POX, APX	CAT	0, 0.15, 0.3, 1.5, 3.0 mM	6	[165]
	SOD, POX, CAT, APX	-	0, 1, 2, 4 mM	3	[166]
	SOD, CAT	APX, GPX, GR	0, 8, 40 mg L^−1^	5	[167]
	SOD	GPX	0, 500, 1000, 2500 μM	7	[168]
*Oryza sativa*	SOD	CAT, POD	0, 50, 100, 200 M	16	[169]
*Triticum aestivum*	SOD, CAT	APX, GPX, GR	0, 50, 100, 250, 500 μM	4	[170]
*Zea mays*	SOD, APX, GPX, GR	CAT	0, 16, 40, 80 mg L^−1^ Pb^2+^	8	[146]
	APX, DHAR, MDHAR	-	0, 16, 40, 80 mg L^−1^ Pb^2+^	1	[171]
*Oryza sativa*	SOD, APX, GR	CAT	0, 10, 50 μM	4	[172]

SOD: Superoxide dismutase; POX: Peroxidase; APX: Ascorbate peroxidase; CAT: Catalase; GPX: Glutathione peroxidise; GR: Glutathione reductase; MDHAR: monodehydroascorbate reductase; DHAR: dehydroascorbate reductase.

**Table 5:** Maximum permissible level of Pb in foodstuffs (mg/kg Fresh Weight).

Lead in Food Stuffs (mg/kg Fresh Weight)	Maximum Permissible Level
Food of Plant Origin	
Rye, grain	0.20
Wheat, grain	0.20
Bread	-
Miscellaneous cereals	-
Cabbage	0.30
Carrot and potatoes	0.10
Apple	0.10
Milk chocolate	-
Food of animal origin	
Carcass meat	0.10
Offal	-
Fish	0.30
Fresh water fish,	0.30
Eggs	-
Milk	0.02
Dairy products	-

**Table 6:** Generalized clinical symptoms of Pb poisoning in humans.

SL No.	Body Organ/System	Clinical Symptoms of Pb Poisoning
1	Eyes	Blindness of parts of visual field
		Hallucinations
2	Ears	Hearing loss
3	Mouth	Unusual taste
		Slurred speech
		Blue line along the gum
4	Kidney	Structural damage and failure
		Changes in the excretory function
5	Liver	Jaundice
		Lead-induced oxidative stress
		Decreased liver function
		Microvesicular and macrovesicular steatosis
		Hemosiderosis and cholestasis
6	Skin	Pallor and/or lividity
7	Central nervous system (CNS)	Insomnia
		Loss of appetite
		Decreased libido
		Depression
		Irritability
		Cognitive deficits
		Memory loss
		Headache
		Personality changes
		Delirium
		Coma
		Encephalopathy
8	Reproductive organs	Sperm dysfunctions
		Pregnancy complications
		Preterm birth
9	Abdomen/Stomach	Pain
		Nausea
		Diarrhoea
		Constipation
10	Blood	Anaemia
11	General	Malaise
		Fatigue
		Weight loss
12	Neuro- muscular	Tremor
		Pain
		Delayed reaction times
		Loss of coordination
		Convulsions
		Foot or ankle drop
		Seizers
		Weakness
13	Bones	Mineralizing bones and teeth
		Decreased bone density

**Table 7:** Chemical amendments for immobilization of lead in soil and groundwater at the field scale [183].

SN	Amendments	Immobilization Mechanisms	Observations	Reference
1.	Clay minerals			
	Sepiolite + limestone	Chemical precipitation and surface complexation	The treatment decreased exchangeable Pb (99.8%) and reduced Pb in brown rice (81.2%).	[202]
			The treatment significantly increased soil pH and CEC, decreased Pb exchangeable fractions, and inhibited Pb accumulation in rice.	[203]
	Palygorskite		Significantly reduced water leachable Pb fractions (50%).	[204]
	Bentonite		Reduced Pb exchangeable fractions (20.3–49.3%). Increased residual portions (6.73–10.0%). Pb concentrations in the rice roots (5.13–26.7%) and shoot (3.73–7.8%) were reduced.	[205]
2.	Phosphate compounds			
	Phosphate rock (Ca_10_(PO_4_)6Cl_2_ Calcium magnesium phosphate (Ca_3_(PO_4_)_2_) Single superphosphate (Ca(H_2_PO_4_)_2_)	Pb: Pb phosphate precipitation, especially pyromorphite-like mineral;	P fertilizers decreased water soluble and exchangeable Pb fractions (22.03–81.4%) and reduced Pb uptakes (16.03–58.0%) by a Chinese green vegetable.	[206]
3.	Liming materials			
	Oyster shells and egg shells	Chemical precipitation	TCLP-leachable Pb was effectively reduced.	[207]
4.	Organic composts			
	Biosolid	Surface complexation and chemical precipitation	The treatment enhanced soil pH, cation exchange capacity, and humic acids, with improved soil sorption capacity. The readily soluble Pb forms were reduced.	[208]
5.	Metal oxides			
	Ferric oxyhydroxide powder + limestone	Specific sorption, co-precipitation, and inner-sphere complex	Pb decreased by 97% in pore water. Pb was transformed into residual mineral.	[209]
	Mn oxides		Pb immobilization.	[210]
6.	Biochar			
	Wheat Straw Biochar	Increase in soil pH, total organic carbon, abundant functional groups, and complex structures of biochar leads to reduction in heavy metals extractable fractions	The soil extractable Pb was decreased. As a result, Pb in root tissues was significantly reduced.	[211]
			Biochar significantly transformed the exchangeable Pb fractions into relatively stable fractions.	[212]
	Sugarcane bagasse biochar		The exchangeable Pb was reduced and the organically-bound fraction increased with increased biochar input. Pb bioavailability to plant shoots and roots decreased with increasing biochar input.	[213]
	Holm oak chips biochar		Biochar stabilized Pb and reduced its accumulation in barley grain.	[214]
	Rice straw biochar		Rice straw biochar decreased. Pb bioavailability and reduced Pb contents in vegetables.	[215]
	Miscanthus (Miscanthus giganteus) straw biochar		CaCl_2_-extractability of Pb significantly decreased with increased biochar input.	[216]

**Table 8:** Phytoremediation potential of different plant species for Pb contaminated water and soil [21].

Species	Treatments	Observation	Findings	References
*Ceratophyllum demersum*	Artificial wastewater	Removal rate 92.0–95.0%	Maximum BCF of 1284.35 in 4 mg/L of Pb 12th Day.	[238]
*Leptodictyum riparium*	Artificial wastewater	Removal rate 96.7%	Having high resistance and effectiveness for Pb accumulation.	[239]
*Scirpus grossus*	600 L spiked water in Pb (10, 30, and 50 mg/L), duration 98 days	Pb concentration in water decreased up to 99% after 28 days and highest Pb uptake: 1343, 4909, 3236 mg/kg for the treatment of 10, 30, and 50 mg/L Pb, respectively	Highest BCF and TF were 485, 261, and 2.52 on day 42 of Pb treatment at 30 mg/L concentration in 70 days retention time.	[240]
*Pistia stratiotes*	Greenhouse condition using glass pots with a defined amount of added HMs	Pb removal was >90% in the first week	No enhancement of Pb removal efficiency with increased Pb concentrations.	[241]
*Eichhornia crassipes*	Operation in up-flow anaerobic packed bed reactors system	Pb Removal rate: 98%	In the coupled pond system, water hyacinth was observed to have enhanced Pb removal efficiency by accumulating Pb into root	[242,243]
*Eichhornia crassipes*	Stock solutions with initial concentration of 20 g/L	Pb Removal rate: 98.33%	Powdered root of water hyacinth absorbed higher Pb.	[244,245]
*Brassica oleracea var.* *Acephala*	Treatments of different Concentration Pb = 0, 1, 5, and 10 mg/kg	Phytoremediation of saline soils with 10 and 16 mg/kg Pb	Negatively correlated with plant fresh and dry weights.	[246]
*Posidonia oceanica*	Sediments	Pb Levels (mg/kg) in root: 4.52 ± 0.55,	Ability of *Posidonia oceanic* to accumulate and detoxify Pb rather than being attributed to differences in ecological and morpho-anatomical characteristics.	[247]
*Datura inoxia*	Concentrations of 0.5, 1.0, 3.0, 5.0, 10, 15, 20, 25, 30, 35, 40, 45, 50 mg/L metal	Survival rate = 50%	*Datura* exhibits phytoremediation potential.	[248]
*Magnolia grandiflora*	Soil	Pb Accumulation rate: 63.4%,	Relationship between heavy metal concentrations in soils and washed new and old leaves.	[249]
*Pistia stratiotes*	HMs from steel effluents: 120 g of plant in 10 L effluent	Removal rates: Pb = 70.7%,	*E. crassipes* more efficient than *P. stratiotes.*	[250]
*Lemna* sp.	Artificial by concentration of 2, 5, and 10 mg/L	Pb removal rates by *Lemna gibba*: 60.1% at 2 mg/L at pH 9, 98.1% at 10 mg/L at pH 7,	BCF and metal uptake yield per unit of dry biomass for Pb is 403–738.	[251]
*Pistia stratiotes*	Stock solution (2000 mg/L)	96% removal of Pb(II) from 25 mL of solution in 60 min by only 0.125 g of biomass	Results consistent with the Langmuir model by maximum biosorption capacity of 122.70 mg Pb (II)/g of biomass.	[252]
*Mixture of Typha angustifolia and Limnocharis flava*	Wastewater oxidation pond	Removal rate: Pb = 62.07%	Positive relation between retention time and heavy metal removal.	[253]
*Lemna* sp.	200 g fresh plant in mixed sewage of industrial and municipal effluents	Pb Removal efficiency >80%	BCFs for Pb = 523, indicating that this plant is a moderate accumulator of Pb.	[254]
*Lemna* sp.	Artificial: Pb = 0.25 mg/L	Removal rates: Pb = 36%	Removal efficiency up to 80% at higher metal loading rate where 24 h light and pre-treatment steps required.	[242,243]
*Eichhornia crassipes*	Mining wastewater	Accumulation in leaves (mg/kg): Pb = 3.40–5.06	BCF: Pb = 242–506	[255]
*Mixture of P. australis and T. latifolia*	Urban sewage mixed with industrial effluents	Removal rate: Pb = 61.0 ± 1.2%	-	[256]

**Table 9:** Characteristics of nano-particles in Pb removal [18].

SN	Nano-Particles	Characters	NP Synthesis	Absorbent Dose	Optimum pH	Removal Efficiency	References
1	Iron oxides NPs	Magnetite nanoparticles	Co-precipitation from a mixture of Fe(II) and (III) salts with aqueous NH_3_ and KOH	50 mg/20cm^3^	5.31–9.37	Pb(II)—76–92%	[274]
2	Ferrite nano particles	-	Modified co-precipitation synthesis	0.008	-	Pb(II) up to 38.1%	[275]
3	Activated carbon NPs	High surface area and greater adsorption capacity	-	0.02	2–10	Pb up to 87%	[276]
4	Nano scale zero valentiron (nZVI)	High surface area and cation exchange capacity	Reduction of Fe(II) using borohydride	-	-	-	[277]
5	Starch stabilized zero valent Iron nanoparticles(nZVI-Starch)	Larger surface area for sorption reactions	Chemical reduction method	1 g/kg soil	4.2	100%	[278]
6	Zeolite materials obtained from fly ash	Greater specific area	Hydrothermal process	6.0	5.6–6.6	>80%	[279]
7	Pyromellitic acid dianhydride/N-(3-(trimethoxysilyl) propylethylene diamine(PMDA/TMSPEDA)	Bound heavy metal ions via co-ordinate and electrostatic interactions	Ring opening polymerization and sol-gel reaction	0.01	7	Pd(II)—79.60%	[280]
8	Ag and Zn nanoparticles functionalized cellulose	High catalytic activity, great biocompatibility, high adsorption capacity, high surface-area, reusability, and greater dispersion degree	Co-precipitation method	0.5 and 1.0	5.5	-	[281]
9	ZnO@Chitosancoreshell Nanocomposite (ZOCS)	Hydrophilicity, biocompatibility, biodegradability, non-toxicity, and High adsorption capacity	Direct precipitation followed by thermal decarbonation	0.02	6	Pb(II) up to 99%	[282]
10	ZnO-Fe_3_O_4_ nanocomposites	High adsorption capacity and surface area	Chemical co-precipitation	0.50	5.5	Pb(II) up to 39.2%	[283]

## References

[1] World Health Organisation (WHO) (2010). Action Is Needed on Chemicals of Major Public Health Concern. Public Health Environ..

[2] Arias J.A., Peralta-Videa J.R., Ellzey J.T., Ren M., Viveros M.N., Gardea-Torresdey J.L. (2010). Effects of *Glomus deserticola* inoculation on Prosopis: Enhancing chromium and lead uptake and translocation as confirmed by X-ray mapping, ICP-OES and TEM techniques. Environ. Exp. Bot..

[3] ATSDR Case Estudies in EnvironmentalL Medicine (CSEM) Lead Toxicity. https://www.atsdr.cdc.gov/csem/lead/docs/CSEM-Lead_toxicity_508.pdf.

[4] Pais I., Jones J.B. (1997). The Handbook of Trace Elements.

[5] Assi M.A., Hezmee M.N.M., Haron A.W., Sabri M.Y., Rajion M.A. (2016). The detrimental effects of lead on human and animal health. Vet. World.

[6] IARC Agents Classified by the IARC Monographs. https://monographs.iarc.fr/wp-content/uploads/2019/02/List_of_Classifications.pdf.

[7] EPA-IRIS Lead and Compounds (Inorganic); CASRN 7439-92-1. Integrated Risk Information System (IRIS), Chemical Assessment Summary. https://cfpub.epa.gov/ncea/iris/iris_documents/documents/subst/0277_summary.pdf.

[8] WHO Lead Poisoning and Health. https://www.who.int/newsroom/fact-sheets/detail/lead-poisoning-and-health.

[9] Saba D., Manouchehri N., Besançon S., El Samad O., Bou Khozam R., Nafeh Kassir L., Kassoufe A., Chebibe H., Ouainib N., Cambier P. (2019). Bioaccessibility of lead in Dittrichia viscosa plants and risk assessment of human exposure around a fertilizer industry in Lebanon. J. Environ. Manag..

[10] Sandin G., Peters G.M. (2018). Environmental impact of textile reuse and recycling—A review. J. Clean. Prod..

[11] Cabral-Pinto M.M.S., Inácio M., Neves O., Almeida A.A., Pinto E., Oliveiros B., Ferreira da Silva E.A.F. (2019). Human Health Risk Assessment Due to Agricultural Activities and Crop Consumption in the Surroundingsof an Industrial Area. Expo. Health.

[12] Cabral-Pinto M.M.S., Ordens C.M., de Melo M.T.C., Inácio M., Almeida A., Pinto E., da Silva E.A.F. (2019). An Inter-disciplinary Approach to Evaluate Human Health Risks Due to Long-Term Exposure to Contaminated Groundwater Near a Chemical Complex. Expo. Health.

[13] Cabral Pinto M., Ferreira da Silva E. (2019). Heavy Metals of Santiago Island (Cape Verde) alluvial deposits: Baseline value maps and human health risk assessment. Int. J. Environ. Res. Public Health.

[14] Lee J.W., Choi H., Hwang U.K., Kang J.C., Kang Y.J., Kim K., Kim J.H. (2019). Toxic effects of lead exposure on bioaccumulation, oxidative stress, neurotoxicity, and immune responses in fish: A review. Environ. Toxi.Pharma..

[15] Hindarwati Y., Soeprobowati T.R., Sudarno (2018). Heavy metal content in terraced rice fieldsat Sruwen Tengaran Semarang—Indonesia. E3S Web Conf..

[16] Zulfiqar U., Farooq M., Hussain S., Maqsood M., Hussain M., Ishfaq M., Ahmad M., Anjum M.J. (2019). Lead toxicity in plants: Impacts and remediation. J. Environ. Manag..

[17] Kumar A., Chaturvedi A.K., Yadav K., kumar K.P., Malyan S.K., Raja P., Kumar R., Khan S.A., Yadav K.K., Rana K.L., Yadav A., Singh S., Mishra S., Gupta A. (2019). Fungal Phytoremediation of Heavy Metal-Contaminated Resources: Current Scenario and Future Prospects. Recent Advancement in White Biotechnology through Fungi. Fungal Biology.

[18] Kumar S., Prasad S., Yadav K.K., Shrivastava M., Gupta N., Nagar S., Bach Q.V., Kamyab H., Khan S.A., Yadav S. (2019). Hazardous heavy metals contamination of vegetables and food chain: Role of sustainable remediation approaches—A review. Environ. Res..

[19] Kumar A., Prasad M.N.V. (2019). Plant genetic engineering approach for the Pb and Zn remediation: Defense reactions and detoxification mechanisms. Transgenic Plant Technology for Remediation of Toxic Metals and Metalloids.

[20] Gupta N., Yadav K.K., Kumar V., Kumar S., Chadd R.P., Kumar A. (2019). Trace elements in soil-vegetables interface: Translocation, bioaccumulation, toxicity and amelioration: A review. Sci. Total Environ..

[21] Yadav K.K., Gupta N., Kumar A., Reece L.M., Singh N., Rezania S., Khan S.A. (2018). Mechanistic understanding and holistic approach of phytoremediation: A review on application and future prospects. Ecol. Eng..

[22] Sammut M., Noack Y., Rose J., Hazemann J., Proux O., Depoux Ziebel M., Fiani E. (2010). Speciation of Cd and Pb in dust emitted from sinter plant. Chemosphere.

[23] Vega F., Andrade M., Covelo E. (2010). Influence of soil properties on the sorption and retention of cadmium, copper and lead, separately and together, by 20 soil horizons: Comparison of linear regression and tree regression analyses. J. Hazard. Mater..

[24] Cecchi M., Dumat C., Alric A., Felix-Faure B., Pradere P., Guiresse M. (2008). Multi-metal contamination of a calcic cambisol by fallout from a lead-recycling plant. Geoderma.

[25] Punamiya P., Datta R., Sarkar D., Barber S., Patel M., Das P. (2010). Symbiotic role of glomus mosseae in phytoextraction of lead in vetiver grass. J. Hazard. Mater..

[26] Ratul A.K., Hassan M., Uddin M.K., Sultana M.S., Akbor M.A., Ahsan M.A. (2018). Potential health risk of heavy metals accumulation in vegetables irrigated with polluted riverwater. Int. Food Res. J..

[27] Chaoua S., Boussaa S., El Gharmali A., Boumezzough A. (2018). Impact of irrigation withwastewater on accumulation of heavy metals in soil and crops in the region of Marrakech inMorocco. J. Saudi Soc. Agric. Sci..

[28] Yang J., Ma S., Zhou J., Song Y., Li F. (2018). Heavy metal contamination in soils andvegetables and health risk assessment of inhabitants in Daye, China. J. Int. Med. Res..

[29] Rahimi G., Kolahchi Z., Charkhabi A. (2017). Uptake and translocation of some heavy metalsby rice crop (*oryza sativa*) in paddy soils. Agriculture.

[30] Showqi I., Lone F.A., Naikoo M. (2018). Preliminary assessment of heavy metals in water, sediment and macrophyte (*Lemna minor*) collected from Anchar Lake, Kashmir, India. Appl. Water Sci..

[31] Sawut R., Kasima N., Maihemuti B., Hue L., Abliz A., Abdujappar A., Kurbana M. (2018). Pollution characteristics and health risk assessment of heavy metals in the vegetable bases ofnorthwest China. Sci. Total Environ..

[32] Latif A., Bilal M., Asghar W., Azeem M., Ahmad M.I. (2018). Heavy metal accumulation invegetables and assessment of their potential health risk. J. Environ. Anal. Chem..

[33] Kumar V., Chopra A.K. (2015). Heavy metals accumulation in soil and agricultural crops grown in the province of Asahi India Glass Ltd., Haridwar (Uttarakhand), India. Adv. Crop Sci. Technol..

[34] Shahid M., Pinelli E., Pourrut B., Silvestre J., Dumat C. (2011). Lead-induced genotoxicity to *Vicia faba* L. roots in relation with metal cell uptake and initial speciation. Ecotoxicol. Environ. Saf..

[35] Tabelin C., Igarashi T. (2009). Mechanisms of arsenic and lead release from hydrothermally altered rock. J. Hazard. Mater..

[36] Lawal O., Sanni A., Ajayi I., Rabiu O. (2010). Equilibrium, thermodynamic and kinetic studies for the biosorption of aqueous lead(II) ions onto the seed husk of *Calophyllum inophyllum*. J. Hazard. Mater..

[37] Bi X., Ren L., Gong M., He Y., Wang L., Ma Z. (2010). Transfer of cadmium and lead from soil to mangoes in an uncontaminated area, Hainan Island, China. Geoderma.

[38] Cenkci S., Cigerci I.H., Yildiz M., Ozay C., Bozdag A., Terzi H. (2010). Lead contamination reduces chlorophyll biosynthesis and genomic template stability in *Brassica rapa* L.. Environ. Exp. Bot..

[39] Padmavathiamma P.K., Li L.Y. (2010). Phytoavailability and fractionation of lead and manganese in a contaminated soil after application of three amendments. Bioresour. Technol..

[40] Kopittke P.M., Asher C.J., Kopittke R.A., Menzies N.W. (2008). Prediction of Pb speciation in concentrated and dilute nutrient solutions. Environ. Pollut..

[41] Komjarova I., Blust R. (2009). Effect of Na, Ca and pH on simultaneous uptake of Cd, Cu, Ni, Pb, and Zn in the water flea *Daphnia magna* measured using stable isotopes. Aquat. Toxicol..

[42] Mager E.M., Esbaugh A.J., Brix K.V., Ryan A.C., Grosell M. (2011). Influences of water chemistry on the acute toxicity of lead to Pimephales promelas and Ceriodaphnia dubia. Comp. Biochem. Physiol. C Toxicol. Pharmacol..

[43] Levin R., Vieira C.L.Z., Mordarski D.C., Rosenbaum M.H. (2019). Lead seasonality in humans, animals, and the natural environment. Environ. Res..

[44] Esbaugh A.J., Brix K.V., Mager E.M., De Schamphelaere K., Grosell M. (2012). Multilinear regression analysis preliminary biotic ligand modeling, and cross species comparison of the effects of water chemistry on chronic lead toxicity in invertebrates. Comp. Biochem. Physiol. C Toxicol. Pharmacol..

[45] Kamel M.M., Ibrahm M.A., Ismael A.M., Motaleeb M.A.E. (2004). Adsorption of some heavy metal ions from aqueous solutions by using kaolinite clay. Ass. Univ. Bull. Environ. Res..

[46] Suzuki T., Niinae M., Koga T., Akita T., Ohta M., Choso T. (2014). EDDS-enhanced electrokinetic remediation of heavy metal-contaminated clay soils under neutral pH conditions. Colloids Surf. Physicochem. Eng. Asp..

[47] Mao L.C., Bailey E.H., Chester J., Dean J., Ander E.L., Chenery S.R., Young S.D. (2014). Lability of Pb in soil: Effects of soil properties and contaminant source. Environ. Chem..

[48] Li X., Meng D., Li J., Yin H., Liu H., Liu X. (2017). Response of soil microbial communities and microbial interactions to long-term heavy metal contamination. Environ. Pollut..

[49] Kögel-Knabner W., Amelung Z., Cao S., Fiedler P., Frenzel R. (2010). Biogeochemistry of paddy soils. Geoderma.

[50] O’Reilly S.E., Hochella M.F. (2003). Lead sorption efficiencies of natural and synthetic Mn and Fe-oxides. Geochem. Cosmochim. Acta.

[51] Tao J., Liu X., Liang Y., Niu J., Xiao Y., Gu Y. (2016). Maize growth responses to soil microbes and soil properties after fertilization with different green manures. Appl. Microbiol. Biotechnol..

[52] Xu X., Hui D., King A.W., Song X., Thornton P.E., Zhang L. (2015). Convergence of microbial assimilations of soil carbon, nitrogen, phosphorus, and sulfur in terrestrial ecosystems. Sci. Rep..

[53] Frank J.J., Poulakos A.G., Tornero-Velez R., Xue J. (2019). Systematic review and meta-analyses of lead (Pb) concentrations in environmental media (soil, dust, water, food, and air) reported in the United States from 1996 to 2016. Sci. Tot. Envir..

[54] Zhao F.J., Ma Y.B., Zhu Y.G., Tang Z., McGrath S.P. (2015). Soil contamination in China: Current status and mitigation strategies. Environ. Sci. Technol..

[55] Emsley J. (2011). Nature’s Building Blocks: An AZ Guide to the Elements.

[56] CDC (2012). Advisory Committee on Childhood Lead Poisoning Prevention (ACCLPP).

[57] United States Environmental Protection Agency (USEPA) (2007). Estimation of Relative Bioavailability of Lead in Soil and Soil-Like Materials Using In Vivo and In Vitro Methods.

[58] Yan K., Dong Z., Wijayawardena M.A.A., Liu Y., Naidu R., Semple K. (2017). Measurement of soil lead bioavailability and influence of soil types and properties: A review. Chemosphere.

[59] Denys S., Caboche J., Tack K., Rychen G., Wragg J., Cave M., Jondreville C., Feidt C. (2012). In Vivo Validation of the Unified BARGE Method to Assess the Bioaccessibility of Arsenic, Antimony, Cadmium, and Lead in Soils. Environ. Sci. Technol..

[60] Deshommes E., Tardif R., Edwards M., Sauvé S., Prevost M. (2012). Experimental determination of the oral bioavailability and bioaccessibility of lead particles. Chem. Cent. J..

[61] Jiang W., Liu D. (2010). Pb-induced cellular defense system in the root meristematic cells of *Allium sativum* L.. BMC Plant Biol..

[62] Yan Z.Z., Ke L., Tam N.F.Y. (2010). Lead stress in seedlings of *Avicennia marina*, a common mangrove species in South China, with and without cotyledons. Aquat. Bot..

[63] Piechalak A., Tomaszewska B., Baralkiewicz D., Malecka A. (2002). Accumulation and detoxification of lead ions in legumes. Phytochemistry.

[64] Małecka A., Piechalak A., Morkunas I., Tomaszewska B. (2008). Accumulation of lead in root cells of Pisum sativum. Acta Physiol. Plant..

[65] Brunet J., Varrault G., Zuily-Fodil Y., Repellin A. (2009). Accumulation of lead in the roots of grass pea (*Lathyrus sativus* L.) plants triggers systemic variation in gene expression in the shoots. Chemosphere.

[66] Gichner T., Znidar I., Száková J. (2008). Evaluation of DNA damage and mutagenicity induced by lead in tobacco plants. Mutat. Res. Genet. Toxicol. Environ. Mutagen..

[67] Gupta D., Huang H., Yang X., Razafindrabe B., Inouhe M. (2010). The detoxification of lead in Sedum alfredii H. is not related to phytochelatins but the glutathione. J. Hazard. Mater..

[68] Kopittke P.M., Asher C.J., Kopittke R.A., Menzies N.W. (2007). Toxic effects of Pb^2+^ on growth of cowpea (Vigna unguiculata). Environ. Pollut..

[69] Metanat K., Ghasemi-Fasaei R., Ronaghi A., Yasrebi J. (2019). Lead Phytostabilization and Cationic Micronutrient Uptake by Maize as Influenced by Pb Levels and Application of Low Molecular Weight Organic Acids. Commun. Soil Sci. Plant Anal..

[70] Meyers D.E.R., Auchterlonie G.J., Webb R.I., Wood B. (2008). Uptake and localisation of lead in the root system of Brassica juncea. Environ. Pollut..

[71] Ginn B.R., Szymanowski J.S., Fein J.B. (2008). Metal and proton binding onto the roots of Fescue rubra. Chem Geol..

[72] Krzeslowska M., Lenartowska M., Mellerowicz E.J., Samardakiewicz S., Wozny A. (2009). Pectinous cell wall thickenings formation–a response of moss protonemata cells to lead. Environ. Exp. Bot..

[73] Krzesłowska M., Lenartowska M., Samardakiewicz S., Bilski H., Wozny A. (2010). Lead deposited in the cell wall of Funaria hygrometrica protonemata is not stable–a remobilization can occur. Environ. Pollut..

[74] Uzu G., Sobanska S., Sarret G., Munoz M., Dumat C. (2010). Foliar lead uptake by lettuce exposed to atmospheric fallouts. Environ. Sci. Technol..

[75] De Forest D.K., Brix K.V., Adams W.J. (2007). Assessing metal bioaccumulation in aquatic environments: The inverse relationship between bioaccumulation factors, trophic transfer factors and exposure concentration. Aquat. Toxicol..

[76] Bhatia A., Singh S., Kumar A. (2015). Heavy metal contamination of soil, irrigation water and vegetables in peri-urban agricultural areas and markets of Delhi. Water Environ. Res..

[77] Jan F.A., Ishaq M., Khan S. (2010). A comparative study of human health risks via consumptionof food crops grown on wastewater irrigated soil (Peshawar) and relatively clean waterirrigated soil (lower Dir). J. Hazard. Mater..

[78] Balkhair K.S., Ashraf M.A. (2016). Field accumulation risks of heavy metals in soil andvegetable crop irrigated with sewage water in western region of Saudi Arabia. Saudi J. Biol. Sci..

[79] Maurya P.K., Malik D.S., Yadav K.K., Kumar A., Kumar S., Kamyab H. (2019). Bioaccumulation and potential sources of heavy metal contamination in fish species in River Ganga basin: Possible human health risks evaluation. Toxicol. Rep..

[80] Chalkiadaki O., Dassenakis M., Lydakis-Simantiris N. (2014). Bioconcentration of Cd and Ni invarious tissues of two marine bivalves living in different habitats and exposed to heavilypolluted seawater. Chem. Ecol..

[81] Ziyaadini M., Yousefiyanpour Z., Ghasemzadeh J. (2017). Biota-sediment accumulation factorand concentration of heavy metals (Hg, Cd, As, Ni, Pb and Cu) in sediments and tissues ofChiton lamyi (Mollusca: Polyplacophora: Chitonidae) in Chabahar Bay, Iran. Iran. J. Fish. Sci..

[82] Yarsan E., Yipel M. (2013). The important terms of marine pollution biomarkers andbiomonitoring, bioaccumulation, bioconcentration, biomagnification. J. Mol. Biomark. Diagn..

[83] Conder J.M., Gobas F.A.P.C., Borga K. (2012). Use of trophic magnification factors and relatedmeasures to characterize bioaccumulation potential of chemicals. Integr. Environ. Assess. Manag..

[84] Zhang C., Wang X., Ashraf U., Qiu B., Ali S. (2017). Transfer of lead (Pb) in the soil-plant-mealybug-ladybird beetle food chain, a comparison between two host plants. Ecotoxicol. Environ. Saf..

[85] Xiong Z., Zhao F., Li M. (2006). Lead toxicity in Brassica pekinensis Rupr: Effect on nitrate assimilation and growth. Environ. Toxicol..

[86] Arshad M., Silvestre J., Pinelli E., Kallerhoff J., Kaemmerer M., Tarigo A., Shahid M., Guiresse M., Pradere P., Dumat C. (2008). A field study of lead phytoextraction by various scented Pelargonium cultivars. Chemosphere.

[87] Maestri E., Marmiroli M., Visioli G., Marmiroli N. (2010). Metal tolerance and hyperaccumulation: Costs and trade-offs between traits and environment. Environ. Exp. Bot..

[88] Liu W., Zhou Q., Zhang Y., Wei S. (2010). Lead accumulation in different Chinese cabbage cultivars and screening for pollution-safe cultivars. J. Environ. Manag..

[89] Verbruggen N., Hermans C., Schat H. (2009). Molecular mechanisms of metal hyperaccumulation in plants. New Phytol..

[90] Liao Y., Chien S.C., Wang M., Shen Y., Hung P., Das B. (2006). Effect of transpiration on Pb uptake by lettuce and on water soluble low molecular weight organic acids in rhizosphere. Chemosphere.

[91] Wang H., Shan X., Wen B., Owens G., Fang J., Zhang S. (2007). Effect of indole-3-acetic acid on lead accumulation in maize (*Zea mays* L.) seedlings and the relevant antioxidant response. Environ. Exp. Bot..

[92] Kim Y.Y., Yang Y.Y., Lee Y. (2002). Pb and Cd uptake in rice roots. Physiol. Plant..

[93] Pourrut B., Perchet G., Silvestre J., Cecchi M., Guiresse M., Pinelli E. (2008). Potential role of NADPH-oxidase in early steps of lead-induced oxidative burst in Vicia faba roots. J. Plant Physiol..

[94] Wojas S., Ruszczynska A., Bulska E., Wojciechowski M., Antosiewicz D.M. (2007). Ca^2+^-dependent plant response to Pb^2+^ is regulated by LCT1. Environ. Pollut..

[95] Sonawane V.Y. (2015). Analysis of Heavy metals in vegetables collected from selected area around Dhulia, North Maharashtra, Maharashtra, India. Analysis.

[96] Jolly Y.N., Islam A., Akbar S. (2013). Transfer of metals from soil to vegetables and possible health risk assessment. SpringerPlus.

[97] Ali M.H., Al-Qahtani K.M. (2012). Assessment of some heavy metals in vegetables, cereals and fruits in Saudi Arabian markets. Egypt. J. Aquat. Res..

[98] Labhade K.R. (2013). Assessment of heavy metal contamination in vegetables grown in and around Nashik City, Maharashtra State, India. IOSR J. Appl. Chem..

[99] Mohod C.V. (2015). A review on the concentration of the heavy metals in vegetable samples like spinach and tomato grown near the area of AmbaNalla of Amravati City. Int. J. Innov. Res. Sci. Eng..

[100] Maleki A., Amini H., Nazmara S., Zandi S., Mahvi A.H. (2014). Spatial distribution of heavy metals in soil, water, and vegetables of farms in Sanandaj, Kurdistan, Iran. J. Environ. Health Sci. Eng..

[101] Gupta S., Jena V., Jena S., Davić N., Matić N., Radojević D., Solanki J.S. (2013). Assessment of heavy metal contents of green leafy vegetables. Croat. J. Food Sci. Technol..

[102] Ramesh H.L., YoganandaMoorthy V.N. (2012). Assessment of heavy metal contamination in green leafy vegetables grown in Bangalore urban district of Karnataka. Adv. Life Sci. Technol..

[103] Ramteke S., Sahu B.L., Dahariya N.S., Patel K.S., Blazhev B., Matini L. (2016). Heavy metal contamination of vegetables. J. Environ. Prot..

[104] Guerra F., Trevizam A.R., Muraoka T., Marcante N.C., Canniatti-Brazaca S.G. (2012). Heavy metals in vegetables and potential risk for human health. Sci. Agric..

[105] Anwar S., Nawaz M.F., Gul S., Rizwan M., Ali S., Kareem A. (2016). Uptake and distribution of minerals and heavy metals in commonly grown leafy vegetable species irrigated with sewage water. Environ. Monit. Assess..

[106] Gebrekidan A., Weldegebriel Y., Hadera A., Van der Bruggen B. (2013). Toxicological assessment of heavy metals accumulated in vegetables and fruits grown in Ginfelriver near Sheba Tannery, Tigray, Northern Ethiopia. Ecotoxicol. Environ. Saf..

[107] Deribachew B., Amde M., Nigussie-Dechassa R., Taddese A.M. (2015). Selected heavy metals in some vegetables produced through wastewater irrigation and their toxicological implications in Eastern Ethiopia. Afr. J. Food Agric. Nutr. Dev..

[108] Mohammed N.K., Khamis F.O. (2012). Assessment of heavy metal contamination in vegetables consumed in Zanzibars. Nat. Sci..

[109] Priya E.S., Sunil G., Shivaiah K., Gaddameedi A., Kumar A. (2014). Extent of heavy metal contamination in leafy vegetables, soil and water from surrounding of Musi river, Hyderabad, India. J. Ind. Pollut. Control..

[110] Adedokun A.H., Njoku K.L., Akinola M.O., Adesuyi A.A., Jolaoso A.O. (2016). Potential human health risk assessment of heavy metals intake via consumption of some leafy vegetables obtained from four market in Lagos metropolis, Nigeria. J. Appl. Sci. Environ. Manag..

[111] Taghipour H., Mosaferi M. (2013). Heavy metals in the vegetables collected from production sites. Health Promot. Perspect..

[112] Soloman P.E., Jain S., Chauhan S.S. (2017). Bio accretion of heavy metals by Okra and Eggplant grown in polluted areas of Jaipur City and associated health risks. Int. J. Innov. Res. Sci. Eng. Technol..

[113] Islam M.S., Hoque M.F. (2014). Concentrations of heavy metals in vegetables around the industrial area of Dhaka city, Bangladesh and health risk assessment. Int. Food Res. J..

[114] Jawad I. (2016). Determination of Heavy Met Herbs Available on the Iraq. Adv. Environ. Biol..

[115] Matloob M.H. (2016). Using Stripping Voltammetry to Determine Heavy Metals in Cooking Spices Used in Iraq. Pol. J. Environ. Stud..

[116] Inam F., Deo S., Narkhede N. (2013). Analysis of minerals and heavy metals in some spices collected from local market. J. Pharaceut. Biol. Sci..

[117] Gaya U.I., Ikechukwu S.A. (2016). Heavy metal contamination of selected spices obtained from Nigeria. J. Appl. Sci. Environ. Manag..

[118] Nkansah M.A., Amoako C.O. (2010). Heavy metal content of some common spices available in markets in the Kumasi metropolis of Ghana. Am. J. Sci. Ind. Res..

[119] Esetlili B.Ç., Pekcan T., Çobanoğlu Ö., Aydoğdu E., Turan S., Anac D. (2014). Essential plant nutrients and heavy metals concentrations of some medicinal and aromatic plants. J. Agric. Sci..

[120] Mosleh Y.Y., Mofeed J., Almaghrabi O.A., Kadasa N.M., El-Alzahrani H.S., Fuller M.P. (2014). Residues of heavy metals, PCDDs, PCDFs, and DL-PCBs some medicinal plants collected randomly from the Jeddah, central market. Life Sci. J..

[121] Ogunkunle A.T.J., Bello O.S., Ojofeitimi O.S. (2014). Determination of heavy metal contamination of street-vended fruits and vegetables in Lagos state, Nigeria. Int. Food Res. J..

[122] Shaheen N., Irfan N.M., Khan I.N., Islam S., Islam M.S., Ahmed M.K. (2016). Presence of heavy metals in fruits and vegetables: Health risk implications in Bangladesh. Chemosphere.

[123] Ibraheen L.H., Abed S.A. (2017). Accumulation detection of some heavy metals in some types of fruits in the local market of Al-Diwaniyah City, Iraq. Rasayan J. Chem..

[124] Radwan M.A., Salama A.K. (2006). Market basket survey for some heavy metals in Egyptian fruits and vegetables. Food Chem. Toxicol..

[125] Sobukola O.P., Adeniran O.M., Odedairo A.A., Kajihausa O.E. (2010). Heavy metal levels of some fruits and leafy vegetables from selected markets in Lagos, Nigeria. Afr. J. Food Sci..

[126] Dahiru M.F., Umar A.B., Sani M.D. (2013). Cadmium, copper, lead and zinc levels in sorghum and millet grown in the city of Kano and its environs. Glob. Adv. Res. J. Environ. Sci. Toxicol..

[127] Guo G., Lei M., Wang Y., Song B., Yang J. (2018). Accumulation of As, Cd, and Pb in sixteen wheat cultivars grown in contaminated soils and associated health risk assessment. Int. J. Environ. Res. Public Health.

[128] Rezapour S., Atashpaz B., Moghaddam S.S., Damalas C.A. (2018). Heavy metal bioavailability and accumulation in winter wheat (*Triticum aestivum* L.) irrigated with treated wastewater in calcareous soils. Sci. Total Environ..

[129] Sakizadeh M., Ghorbani H. (2017). Concentration of heavy metals in soil and staple crops and the associated health risk. Arch. Hyg. Sci..

[130] Haseeb M., Basra S.M.A., Afzal I., Wahid A. (2018). Quinoa response to lead: Growth and lead partitioning. Int. J. Agric. Biol..

[131] Kacalkova L., Tlustos P., Szakova J. (2014). Chromium, nickel, cadmium, and lead accumulation in maize, sunflower, willow, and poplar. Pol. J. Environ. Stud..

[132] Akenga T., Sudoi V., Machuka W., Kerich E., Ronoh E. (2017). Heavy metals uptake in maize grains and leaves in different agro ecological zones in Uasin Gishu County. J. Environ. Prot..

[133] Islam M.S., Ahmed M.K., Habibullah-Al-Mamun M. (2014). Heavy metals in cereals and pulses: Health implications in Bangladesh. J. Agric. Food Chem..

[134] Lee K.J., Feng Y.Y., Choi D.H., Lee B.W. (2016). Lead accumulation and distribution in different rice cultivars. J. Crop Sci. Biotechnol..

[135] Ihedioha J.N., Ujam O.T., Nwuche C.O., Ekere N.R., Chime C.C. (2016). Assessment of heavy metal contamination of rice grains (Oryza sativa) and soil from Ada field, Enugu, Nigeria: Estimating the human health risk. Hum. Ecol. Risk Assess. Int. J..

[136] Kahraman A., Onder M. (2018). Accumulation of heavy metals in dry beans sown on different dates. J. Elem..

[137] Sadrabad E.K., Boroujeni H.M., Heydari A. (2018). Heavy metal accumulation in soybeans cultivated in Iran, 2015–2016. J. Nutr. Food Secur..

[138] Palizban A., Badii A., Asghari G., Nafchi M. (2015). Lead and cadmium contamination in seeds and oils of Brassica napus L. and Carthamus tinctorius grown in Isfahan province/Iran. Iran. J. Toxicol..

[139] Barbillon A., Aubry C., Nold F., Besancon S., Manouchehri N. (2019). Health Risks Assessment in Three Urban Farms of Paris Region for Different Scenarios of Urban Agricultural Users: A Case of Soil Trace Metals Contamination. J. Agric. Sci..

[140] Liu X., Song Q., Tang Y., Li W., Xu J., Wu J., Fan W., Brookes P.C. (2013). Human health risk assessment of heavy metals in soil–vegetable system: A multi-medium analysis. Sci. Total Environ..

[141] Laidlaw M.A.S., Filippelli G.M., Sadler R.C., Gonzales C.R., Ball A.S., Mielke H.W. (2016). Children’s Blood Lead Seasonality in Flint, Michigan (USA), and Soil-Sourced Lead Hazard Risks. Int. J. Environ. Res. Public Health.

[142] Ngueta G., Abdous B., Tardif R., St-Laurent J., Levallois P. (2016). Use of a cumulative exposure index to estimate the impact of tap water lead concentration on blood lead levels in 1- to 5-year-old children (Montreal, Canada). Environ. Health Perspect..

[143] Zahran S., Laidlaw M.A.S., McElmurry S.P., Filippelli G.M., Taylor M. (2013). Linking Source and Effect: Resuspended Soil Lead, Air Lead, and Children’s Blood Lead Levels in Detroit, Michigan. Environ. Sci. Technol..

[144] Lebov J., Grieger K., Womack D., Zaccaro D., Whitehead N., Kowalcyk B., MacDonald P.D.M. (2017). A framework for One Health research. One Health.

[145] Davisa M.F., Rankin S.C., Schurer J.M., Cole S., Conti L., Rabinowitz P. (2017). COHERE Expert Review Group. Checklist for One Health Epidemiological Reporting of Evidence (COHERE). One Health.

[146] Kaur G., Singh H.P., Batish D.R., Kohli R.K. (2015). Adaptations to oxidative stress in Zea mays roots under short term Pb^2+^ exposure. Biologia.

[147] Rai P.K., Lee S.S., Zhang M., Tsang Y.F., Kim K.H. (2019). Heavy metals in food crops: Health risks, fate, mechanisms, and Management. Environ. Inter..

[148] Ekmekci Y., Tanyolac D., Ayhan B. (2009). A crop tolerating oxidative stress induced by excess lead: Maize. Acta Physiol. Plant..

[149] Kabata-Pendias A., Szteke B. (2015). Trace Elements in Abiotic and Biotic Environments.

[150] World Health Organisation (WHO) Safety evaluation of certain food additives and contaminants. https://apps.who.int/iris/bitstream/handle/10665/44515/WHO_TRS_960_eng.pdf;jsessionid=AFB1D9565016562EA09FFBFF1734B190?sequence=1.

[151] Hsu P.C., Guo Y.L. (2002). Antioxidant nutrients and lead toxicity. Toxicology.

[152] Concha G., Eneroth H., Hallstrom H., Sand S. (2013). Contaminants and Minerals in Foods for Infants and Young Children.

[153] World Health Organisation (WHO) (2010). Childhood Lead Poisoning.

[154] EC. Commission of the European Communities (2006). Commission Regulation (EC) No. 1881/2006 Regulation ofsetting maximum levels for certain contaminants infoodstuffs L364-5/L364-24. Off. J. Eur. Union.

[155] Bellinger D.C., Malin A., Wright R.O. (2018). The Neurodevelopmental Toxicity of Lead: History, Epidemiology, and Public Health Implications. Adv. Neurotoxicol..

[156] Wani A.L., Ara A., Usmani J.A. (2015). Lead toxicity: A review. Interdiscip. Toxicol..

[157] Gao H., Liu C.P., Song S.Q., Fu J. (2016). Effects of Dietary Selenium Against Lead Toxicity on mRNA Levels of 25 Selenoprotein Genes in the Cartilage Tissue of Broiler Chicken. Biol. Trace Elem. Res..

[158] Rahman S., Joshi M.V. (2009). Effect of lead toxicity on growth and performance of broilers. Tamilnadu J. Vet. Anim. Sci..

[159] Flora G., Gupta D., Tiwari A. (2012). Toxicity of lead: A review with recent updates. Interdiscip. Toxicol..

[160] Flora S.J.S. (2002). Nutritional components modify metal absorption, toxic response and chelation therapy. J. Nutr. Environ. Med..

[161] Kasperczyk A., Machnik G., Dobrakowski M., Sypniewski D., Birkner E., Kasperczyk S. (2012). Gene expression and activity of antioxidant enzymes in the blood cells of workers who were occupationally exposed to lead. Toxicology.

[162] Kasperczyk S., Dobrakowski M., Kasperczyk A., Ostałowska A., Birkner E. (2013). The administration of N-acetylcysteine reduces oxidative stress and regulates glutathione metabolism in the blood cells of workers exposed to lead. Clin. Toxicol..

[163] Gangoso L., Lloret P.A., Rodrıguez-Navarro A.A.B., Mateo R., Hiraldo F., Donazar J.A. (2009). Long-term effects of lead poisoning on bone mineralization in vultures exposed to ammunition sources. Environ. Pollut..

[164] Theppeang K., Glass T.A., Bandeen-Roche K., Todd A.C., Rohde C.A., Links J.M., Schwartz B.S. (2008). Associations of Bone Mineral Density and Lead Levels in Blood, Tibia, and Patella in Urban-Dwelling Women. Environ. Health Perspect..

[165] Lamhamdi M., Bakrim A., Aarab A., Lafont R., Sayah F. (2011). Lead phytotoxicity on wheat (*Triticum aestivum* L.) seed germination and seedlings growth. Comptes Rendus Biol..

[166] Yang Y., Zhang Y., Wei X., You J., Wang W., Lu J., Shi R. (2011). Comparative antioxidative responses and proline metabolism in two wheat cultivars under short term lead stress. Ecotoxicol. Environ. Saf..

[167] Kaur G., Singh H.P., Batish D.R., Kohli R.K. (2012). A time course assessment of changes in reactive oxygen species generation and antioxidant defense in hydroponically grown wheat in response to lead ions (Pb^2+^). Protoplasma.

[168] Kaur G., Singh H.P., Batish D.R., Kumar R.K. (2012). Growth, photosynthetic activity and oxidative stress in wheat (Triticum aestivum) after exposure of lead to soil. J. Environ. Biol..

[169] Li X., Bu N., Yueying L., Maa L., Xin S., Zhang L. (2012). Growth, photosynthesis and antioxidant responses of endophyte infected and noninfected rice under lead stress conditions. J. Hazard. Mater..

[170] Kaur G., Singh H.P., Batish D.R., Kohli R.K. (2013). Lead (Pb)-induced biochemical and ultrastructural changes in wheat (Triticum aestivum) roots. Protoplasma.

[171] Kaur G., Kaur S., Singh H.P., Batish D.R., Kohli R.K., Rishi V. (2015). Biochemical adaptations in Zea maysroots to short-term Pb^2+^ exposure: ROS generation and metabolism. Bull. Environ. Contam. Toxicol..

[172] Thakur S., Singh L., Zularisam A.W., Sakinah M., Din M.F.M. (2017). Lead induced oxidative stress and alteration in the activities of antioxidative enzymes in rice shoots. Biol. Plant..

[173] Rumbeiha W.K., Braselton W.E., Donch D. (2001). A retrospective study of the disappearance of blood lead in cattle with accidental lead toxicosis. J. Vet. Diagn. Investig..

[174] Sharpe R.T., Livesey C.T. (2006). Lead poisoning in cattle and its implications for food safety. Vet. Rec..

[175] Abadin H., Ashizawa A., Stevens Y.W., Llados F., Diamond G., Sage G., Quinones A., Bosch S.J., Swarts S.G. (2007). Toxicological Profile for Lead, Atlanta (GA): Agency for Toxic Substances and Disease Registry (US).

[176] Yuan G., Dai S., Yin Z., Lu H., Jia R., Xu J., Song X., Li L., Shu Y., Zhao X. (2014). Toxicological assessment of combined lead and cadmium: Acute and sub-chronic toxicity study in rats. Food Chem. Toxicol..

[177] Cobbina S.J., Chen Y., Zhou Z., Wu X., Zhao T., Zhang Z., Feng W., Wang W., Li Q., Wu X. (2015). Toxicity assessment due to sub-chronic exposure to individual and mixtures of four toxic heavy metals. J. Hazard. Mater..

[178] Shaban El-Neweshy M., Said El-Sayed Y. (2011). Influence of vitamin C supplementation on lead-induced histopathological alterations in male rats. Exp. Toxicol. Pathol..

[179] Abdou H.M., Hassan M.A. (2014). Protective role of omega-3 polyunsaturated fatty acid against lead acetate-induced toxicity in liver and kidney of female rats. BioMed Res. Int..

[180] Carocci A., Catalano A., Lauria G., Sinicropi M.S., Genchi G. (2016). Lead toxicity, antioxidant defense and environment. Rev. Environ. Contam. Toxicol..

[181] International Agency for Research on Cancer (IARC) (2018). Agents Classified by the IARC Monographs, Volumes 1–121.

[182] Bhargava A., Carmona F.F., Bhargava M., Srivastava S. (2012). Approaches for enhanced phytoextraction of heavy metals. J. Environ. Manag..

[183] Gong Y., Zhao D., Wang Q. (2018). An overview of field-scale studies on remediation of soil contaminated with heavy metals and metalloids: Technical progress over the last decade. Water Res..

[184] Mallampati S.R., Mitoma Y., Okuda T., Simion C., Lee B.K. (2015). Dynamic immobilization of simulated radionuclide 133Cs in soil by thermal treatment/vitrification with nanometallic Ca/CaO composites. J. Environ. Radioact..

[185] Dellisanti F., Rossi P.L., Valdre G. (2009). In-field remediation of tons of heavy metal-rich waste by Joule heating vitrification. Int. J. Miner. Process..

[186] Wang Q., Tian S., Wang Q., Huang Q., Yang J. (2008). Melting characteristics during the vitrification of MSWI fly ash with a pilot-scale diesel oil furnace. J. Hazard. Mater..

[187] Navarro A., Cardellach E., Canadas I., Rodríguez J. (2013). Solar thermal vitrification of mining contaminated soils. Int. J. Miner. Process..

[188] Yao Z., Li J., Xie H., Yu C. (2012). Review on remediation technologies of soil contaminated by heavy metals. Proc. Environ. Sci..

[189] Kim W.S., Park G.Y., Kim D.H., Jung H.B., Ko S.H., Baek K. (2012). In situ field scale electrokinetic remediation of multi-metals contaminated paddy soil: Influence of electrode configuration. Electrochim. Acta.

[190] Jeon E.K., Jung J.M., Kim W.S., Ko S.H., Baek K. (2015). In situ electrokinetic remediation of As-, Cu-, and Pb-contaminated paddy soil using hexagonal electrode configuration: A full scale study. Environ. Sci. Pollut. Control Ser..

[191] Khan F.I., Husain T., Hejazi R. (2004). An overview and analysis of site remediation technologies. J. Environ. Manag..

[192] Chen Q.Y., Tyrer M., Hills C.D., Yang X.M., Carey P. (2009). Immobilisation of heavy metal in cement-based solidification/stabilisation: A review. Waste Manag..

[193] Wang F., Wang H., Al-Tabbaa A. (2014). Leachability and heavy metal speciation of 17-year old stabilised/solidified contaminated site soils. J. Hazard. Mater..

[194] Antemir A., Hills C.D., Carey P.J., Magnie M.C., Polettini A. (2010). Investigation of 4-year-old stabilised/solidified and accelerated carbonated contaminated soil. J. Hazard. Mater..

[195] Navarro-Blasco I., Duran A., Sirera R., Fernández J.M., Alvarez J.I. (2013). Solidification/stabilization of toxic metals in calcium aluminate cement matrices. J. Hazard. Mater..

[196] Voglar G.E., Leštan D. (2013). Equilibrium leaching of toxic elements from cement stabilized soil. J. Hazard. Mater..

[197] Wang F., Wang H., Jin F., Al-Tabbaa A. (2015). The performance of blended conventional and novel binders in the in-situ stabilisation/solidification of a contaminated site soil. J. Hazard. Mater..

[198] Ferraro A., Fabbricino M., van Hullebusch E.D., Esposito G., Pirozzi F. (2015). Effect of soil/contamination characteristics and process operational conditions on aminopolycarboxylates enhanced soil washing for heavy metals removal: A review. Rev. Environ. Sci. Biotechnol..

[199] Park B., Son Y. (2017). Ultrasonic and mechanical soil washing processes for the removal of heavy metals from soils. Ultrason. Sonochem..

[200] Hu P., Yang B., Dong C., Chen L., Cao X., Zhao J., Wu L., Luo Y., Christie P. (2014). Assessment of EDTA heap leaching of an agricultural soil highly contaminated with heavy metals. Chemosphere.

[201] Wang G., Zhang S., Zhong Q., Xu X., Li T., Jia Y., Zhang Y., Peijnenburg W.J.G.M., Vijver M.G. (2018). Effect of soil washing with biodegradable chelators on the toxicity of residual metals and soil biological properties. Sci. Total Environ..

[202] Wu Y.J., Zhou H., Zou Z.J., Zhu W., Yang W.T., Peng P.Q., Zeng M., Liao B.H. (2016). A three-year in-situ study on the persistence of a combined amendment (limestone+sepiolite) for remedying paddy soil polluted with heavy metals. Ecotoxicol. Environ. Saf..

[203] Zhou H., Zhou X., Zeng M., Liao B.H., Liu L., Yang W.T., Wu Y.M., Qiu Q.Y., Wang Y.J. (2014). Effects of combined amendments on heavy metal accumulation in rice (*Oryza sativa* L.) planted on contaminated paddy soil. Ecotoxicol. Environ. Saf..

[204] Zotiadis V., Argyraki A., Theologou E. (2012). Pilot-scale application of attapulgitic clay for stabilization of toxic elements in contaminated soil. J. Geotech. Geoenviron. Eng..

[205] Sun Y., Li Y., Xu Y., Liang X., Wang L. (2015). In situ stabilization remediation of cadmium (Cd) and lead (Pb) co-contaminated paddy soil using bentonite. Appl. Clay Sci..

[206] Wang B., Xie Z., Chen J., Jiang J., Su Q. (2008). Effects of field application of phosphate fertilizers on the availability and uptake of lead, zinc and cadmium by cabbage (*Brassica chinensis* L.) in a mining tailing contaminated soil. J. Environ. Sci..

[207] Lim J.E., Ahmad M., Lee S.S., Shope C.L., Hashimoto Y., Kim K.R., Usman A.R.A., Yang J.E., Ok Y.S. (2013). Effects of lime-based waste materials on immobilization and phytoavailability of cadmium and lead in contaminated soil. Clean-Soil Air Water.

[208] Placek A., Grobelak A., Kacprzak M. (2016). Improving the phytoremediation of heavy metals contaminated soil by use of sewage sludge. Int. J. Phytoremediat..

[209] Okkenhaug G., Grasshorn Gebhardt K.A., Amstaetter K., Lassen Bue H., Herzel H., Mariussen E., Rossebø Almås Å., Cornelissen G., Breedveld G.D., Rasmussen G. (2016). Antimony (Sb) and lead (Pb) in contaminated shooting range soils: Sb and Pb mobility and immobilization by iron based sorbents, a field study. J. Hazard. Mater..

[210] McCann C.M., Gray N.D., Tourney J., Davenport R.J., Wade M., Finlay N., Hudson-Edwards K.A., Johnson K.L. (2015). Remediation of a historically Pb contaminated soil using a model natural Mn oxide waste. Chemosphere.

[211] Bian R., Joseph S., Cui L., Pan G., Li L., Liu X., Zhang A., Rutlidge H., Wong S., Chia C. (2014). A three-year experiment confirms continuous immobilization of cadmium and lead in contaminated paddy field with biochar amendment. J. Hazard. Mater..

[212] Cui L., Pan G., Li L., Bian R., Liu X., Yan J., Quan G., Ding C., Chen T., Liu Y. (2016). Continuous immobilization of cadmium and lead in biochar amended contaminated paddy soil: A five-year field experiment. Ecol. Eng..

[213] Nie C., Yang X., Niazi N.K., Xu X., Wen Y., Rinklebe J., Ok Y.S., Xu S., Wang H. (2018). Impact of sugarcane bagasse-derived biochar on heavy metal availability and microbial activity: A field study. Chemosphere.

[214] Moreno-Jimenez E., Fernandez J.M., Puschenreiter M., Williams P.N., Plaza C. (2016). Availability and transfer to grain of As, Cd, Cu, Ni, Pb and Zn in a barley agri-system: Impact of biochar, organic and mineral fertilizers. Agric. Ecosyst. Environ..

[215] Niu L., Jia P., Li S., Kuang J., He X., Zhou W., Liao B., Shu W., Li J. (2015). Slash-and char: An ancient agricultural technique holds new promise for management of soils contaminated by Cd, Pb and Zn. Environ. Pollut..

[216] Houben D., Evrard L., Sonnet P. (2013). Mobility bioavailability and pH-dependent leaching of cadmium, zinc and lead in a contaminated soil amended with biochar. Chemosphere.

[217] Cheng S.F., Huang C.Y., Chen K.L., Lin S.C., Lin Y.C. (2016). Phytoattenuation of lead-contaminated agricultural land using *Miscanthus floridulus*—An in situ case study. Desalin. Water Treat..

[218] Yang J., Yang J., Huang J. (2017). Role of co-planting and chitosan in phytoextraction of as and heavy metals by Pteris vittata and castor bean—A field case. Ecol. Eng..

[219] Basha S.A., Rajaganesh K. (2014). Microbial Bioremediation of Heavy Metals from Textile Industry Dye Effluents using Isolated Bacterial Strains. Int. J. Curr. Microbiol. Appl. Sci..

[220] Kang C.H., So J.S. (2016). Heavy metal and antibiotic resistance of ureolytic bacteria and their immobilization of heavy metals. Ecol. Eng..

[221] Puyen Z.M., Villagrasa E., Maldonado J., Diestra E., Esteve I., Solé A. (2012). Biosorption of lead and copper by heavy-metal tolerant Micrococcus luteus DE2008. Bioresour. Technol..

[222] Jin Y., Luan Y., Ning Y., Wang L. (2018). Effects and mechanisms of microbial remediation of heavy metals in soil: A critical review. Appl. Sci..

[223] Jacob J.M., Karthik C., Saratale R.G., Kumar S.S., Prabakar D., Kadirvelu K., Pugazhendhi A. (2018). Biological approaches to tackle heavy metal pollution: A survey of literature. J. Environ. Manag..

[224] Wang T., Sun H., Mao H., Zhang Y., Wang C., Zhang Z., Wang B., Sun L. (2014). The immobilization of heavy metals in soil by bioaugmentation of a UV-mutant Bacillus subtilis 38 assisted by NovoGro biostimulation and changes of soil microbial community. J. Hazard. Mater..

[225] Zeng X., Wei S., Sun L., Jacques D.A., Tang J., Lian M., Xu Z. (2015). Bioleaching of heavy metals from contaminated sediments by the Aspergillus niger strain SY1. J. Soils Sediments.

[226] Dursun A.Y., Uslu G., Cuci Y., Aksu Z. (2003). Bioaccumulation of copper (II), lead (II) and chromium (VI) by growing Aspergillus niger. Process. Biochem..

[227] Kariuki Z., Kiptoo J., Onyancha D. (2017). Biosorption studies of lead and copper using rogers mushroom biomass Lepiota hystrix. S. Afr. J. Chem. Eng..

[228] Sooksawat N., Meetam M., Kruatrachue M., Pokethitiyook P., Nathalang K. (2013). Phytoremediation potential of charophytes: Bioaccumulation and toxicity studies of cadmium, lead and zinc. J. Environ. Sci..

[229] Ibrahim W.M., Abdel Aziz Y.S., Hamdy S.M., Gad N.S. (2018). Comparative Study for Biosorption of Heavy Metals from Synthetic Wastewater by Different Types of Marine Algae. J. Bioremed. Biodegrad..

[230] Jafari M., Younes R.D., Yubert G. (2013). Molecular Techniques in Fungal Bioremediation. Fungi as Bioremediators.

[231] Subrahmanyam G., Hu H.W., Zheng Y.M., Gattupalli A., He J.Z., Liu Y.R. (2014). Response of ammonia oxidizing microbes to the stresses of arsenic and copper in two acidic alfisols. Appl. Soil Ecol..

[232] Subrahmanyam G., Shen J.P., Liu Y.R., Archana G., Zhang L.M. (2016). Effect of long-term industrial waste effluent pollution on soil enzyme activities and bacterial community composition. Environ. Monit. Assess..

[233] Subrahmanyam G., Sharma R.K., Kumar G.N., Archana G. (2018). *Vigna radiata* var. GM4 plant growth enhancement and root colonization by a multi-metal-resistant plant growth-promoting bacterium *Enterobacter* sp. C1D in Cr (VI)-amended soils. Pedosphere.

[234] Jaiswal S., Singh D.K., Shukla P. (2019). Gene editing and systems biology tools for pesticide bioremediation: A review. Front. Microbiol..

[235] Babu A.G., Kim J.D., Oh B.T. (2013). Enhancement of heavy metal phytoremediation by Alnus firma with endophytic Bacillus thuringiensis GDB1. J. Hazard. Mater..

[236] Sheng X.F., Xia J.J., Jiang C.Y., He L.Y., Qian M. (2008). Characterization of heavy metal-resistant endophytic bacteria from rape (Brassica napus) roots and their potential in promoting the growth and lead accumulation of rape. Environ. Pollut..

[237] Tak H.I., Ahmad F., Babalola O.O., Whitacre D.M. (2013). Advances in the Application of Plant Growth-Promoting Rhizobacteria in Phytoremediation of Heavy Metals. Reviews of Environmental Contamination and Toxicology.

[238] Lima L.K.S., Pelosi B.T., Silva M.G.C., Vieira M.G.A. (2013). Lead and chromium biosorption by Pistia stratiotes biomass. Chem. Eng. Trans..

[239] Basile A., Sorbo S., Conte B., Cobianchi R.C., Trinchella F., Capasso C., Carginale V. (2012). Toxicity, accumulation, and removal of heavy metals by three aquatic macrophytes. Int. J. Phytoremediat..

[240] Tangahu B.V., Abdullah S.R.S., Basri H., Idris M., Anuar N., Mukhlisin M. (2013). Phytoremediation of wastewater containing lead (Pb) in pilot reed bed using *Scirpus grossus*. Int. J. Phytoremediat..

[241] Veselý T., Tlustos P., Száková J. (2011). The use of water lettuce (*Pistia stratiotes* L.) for rhizofiltration of a highly polluted solution by cadmium and lead. Int. J. Phytoremediat..

[242] Sekomo C.B., Rousseau D.P.L., Saleh S.A., Lens P.N.L. (2012). Heavy metal removal in duckweed and algae ponds as a polishing step for textile waste water treatment. Ecol. Eng..

[243] Sekomo C.B., Kagisha V., Rousseau D., Lens P. (2012). Heavy metal removal by combining anaerobic upflow packed bed reactors with water hyacinth ponds. Environ. Technol..

[244] Li Q., Chen B., Lin P., Zhou J., Zhan J., Shen Q., Pan X. (2016). Adsorption of heavy metal from aqueous solution by dehydrated root powder of long-root Eichhornia crassipes. Int. J. Phytoremediat..

[245] Li R., Zhou Z., Xie X., Li Y., Zhang Y., Xu X. (2016). Effects of dissolved organic matter on uptake and translocation of lead in Brassica chinensis and potential health risk of Pb. Int. J. Environ. Res. Public Health.

[246] Haghighi M., Kafi M., Pessarakli M., Sheibanirad A., Sharifinia M.R. (2016). Using kale (Brassica oleracea var. acephala) as a phytoremediation plant species for lead (Pb) and cadmium (Cd) removal in saline soils. J. Plant Nutr..

[247] Bonanno G., Borg J.A., Martino V.D. (2017). Levels of heavy metals in wetland and marine vascular plants and their biomonitoring potential: A comparative assessment. Sci. Total Environ..

[248] Waoo A.A. (2016). Comparative effect of four heavy metals, Cd, Pb, Ni, and Cr, on Datura inoxia in Tissue Culture. Int. J. Adv. Eng. Technol. Sci..

[249] Liang J., Fang H.L., Zhang T.L., Wang X.X., Liu Y.D. (2017). Heavy metal in leaves of twelve plant species from seven different areas in Shanghai, China. Urban For. Urban Green..

[250] Aurangzeb N., Nisa S., Bibi Y., Javed F., Hussain F. (2014). Phytoremediation potential of aquatic herbs from steel foundry effluent. Braz. J. Chem. Eng..

[251] Verma R., Suthar S. (2015). Lead and cadmium removal from water using duckweed-Lemna gibba L.: Impact of pH and initial metal load. Alex. Eng. J..

[252] Volf I., Rakoto N.G., Bulgariu L. (2015). Valorization of Pistia stratiotes biomass as biosorbent for lead (II) ions removal from aqueous media. Sep. Sci. Technol..

[253] Syukor A.R.A., Sulaiman S., Siddique M.N.I., Zularisam A.W., Said M.I.M. (2016). Integration of phytogreen for heavy metal removal from wastewater. J. Clean. Prod..

[254] Bokhari S.H., Ahmad I., Mahmood-Ul-Hassan M., Mohammad A. (2016). Phytoremediation potential of Lemna minor L. for heavy metals. Int. J. Phytoremediat..

[255] Prasad B., Maiti D. (2016). Comparative study of metal uptake by Eichhornia crassipes growing in ponds from mining and nonmining areas—A field study. Bioremediat. J..

[256] Kumari M., Tripathi B.D. (2015). Efficiency of Phragmites australis and Typha latifolia for heavy metal removal from wastewater. Ecotoxicol. Environ. Saf..

[257] Lorenzo-Gutiérrez D., Gómez-Gil L., Guarro J., Roncero M.I.G., Fernández-Bravo A., Capilla J., López-Fernández L. (2019). Role of the Fusarium oxysporum metallothionein Mt1 in resistance to metal toxicity and virulence. Metallomics.

[258] Hattab S., Flores-Casseres M.L., Boussetta H., Doumas P., Hernandez L.E., Banni M. (2016). Characterisation of lead-induced stress molecular biomarkers in Medicago sativa plants. Environ. Exp. Bot..

[259] Fan T., Yang L., Wu X., Ni J., Jiang H., Zhang Q.A., Fang L., Sheng Y., Ren Y., Cao S. (2016). The PSE1 gene modulates lead tolerance in Arabidopsis. J. Exp. Bot..

[260] Jiang L., Wang W., Chen Z., Gao Q., Xu Q., Cao H. (2017). A role for APX1 gene in lead tolerance in Arabidopsis thaliana. Plant Sci..

[261] Kim D.Y., Bovet L., Kushnir S., Noh E.W., Martinoia E., Lee Y. (2006). AtATM3 is involved in heavy metal resistance in Arabidopsis. Plant Physiol..

[262] Jiang Y., Wang W., Xie Q., Liu N., Liu L., Wang D., Zhang X., Yang C., Chen X., Tang D. (2017). Plants transfer lipids to sustain colonization by mutualistic mycorrhizal and parasitic fungi. Science.

[263] Xiao S., Gao W., Chen Q.F., Ramalingam S., Chye M.L. (2008). Overexpression of membrane-associated acyl-CoA-binding protein ACBP1 enhances lead tolerance in Arabidopsis. Plant J..

[264] Cao S., Chen Z., Liu G., Jiang L., Yuan H., Ren G., Bian X.H., Jian H., Ma X. (2009). The Arabidopsis Ethylene-Insensitive 2 gene is required for lead resistance. Plant Physiol. Biochem..

[265] Zhu F.Y., Li L., Lam P.Y., Chen M.X., Chye M.L., Lo C. (2013). Sorghum extracellular leucine-rich repeat protein SbLRR2 mediates lead tolerance in transgenic Arabidopsis. Plant Cell Physiol..

[266] Yang L., Fan T., Guan L., Ren Y., Han Y., Liu Q., Liu Y., Cao S. (2016). CMDH4 encodes a protein that is required for lead tolerance in Arabidopsis. Plant Soil.

[267] Anjum M., Miandad R., Waqas M., Gehany F., Barakat M.A. (2016). Remediation ofwastewater using various nano-materials. Arab. J. Chem..

[268] Estrella-Gomez N., Mendoza-Cozatl D., Moreno-Sanchez R., Gonzalez-Mendoza D., Zapata-Perez O., Martınez-Hernandez A., Santamaria J.M. (2009). The Pb-hyperaccumulator aquatic fern Salvinia minima Baker, responds to Pb21 by increasing phytochelatins via changes in SmPCS expression and in phytochelatin synthase activity. Aquat. Toxicol..

[269] Adeleye A.S., Conway J.R., Garner K., Huang Y., Su Y., Keller A.A. (2016). Engineerednanomaterials for water treatment and remediation: Costs, benefits, and applicability. Chem. Eng. J..

[270] Wernisch S., Trapp O., Lindner W. (2013). Application of cinchona-sulfonate-based chiralzwitterionic ion exchangers for the separation of proline-containing dipeptide rotamers anddetermination of on-column isomerization parameters from dynamic elution profiles. Anal. Chim. Acta.

[271] Yong-Mei H., Man C., Zhong-Bo H. (2010). Effective removal of Cu (II) ions from aqueoussolution by amino-functionalized magnetic nanoparticles. J. Hazard. Mater..

[272] Yadav K.K., Singh J.K., Gupta N., Kumar V. (2017). A review of nano-bioremediationtechnologies for environmental cleanup: A novel biological approach. J. Mater. Environ. Sci..

[273] Srivastav A., Yadav K.K., Yadav S., Gupta N., Singh J.K., Katiyar R., Kumar V. (2018). Nano-phytoremediation of pollutants from contaminated soil environment: Current scenario and future prospects. Phytoremediation.

[274] Bobik M., Korus I., Dudek L. (2017). The effect of magnetite nanoparticles synthesis conditionson their ability to separate heavy metal ions. Arch. Environ. Prot..

[275] Klekotka U., Wińska E., Zambrzycka-Szelewa E., Satuła D., Kalska-Szostko B. (2018). Heavy-metal detectors based on modified ferrite nanoparticles. Beilstein J. Nanotechnol..

[276] Hegazi H.A. (2013). Removal of heavy metals from wastewater using agricultural and industrialwastes as adsorbents. HBRC J..

[277] Mohammed A.A., Brouers F., Sadi S.I.A., Al-Musawi T.J. (2018). Role of Fe_3_O_4_ magnetitenanoparticles used to coat bentonite in zinc (II) ions sequestration. Environ. Nanotechnol. Monit. Manag..

[278] Okuo J., Emina A., Stanley O., Anegbe B. (2018). Synthesis, characterization and applicationof starch stabilized zerovalent iron nanoparticles in the remediation of Pb-acid battery soil. Environ. Nanotechnol. Monit. Manag..

[279] Visa M. (2016). Synthesis and characterization of new zeolitematerials obtained from fly ash forheavy metals removal inadvanced wastewater treatment. Powder Technol..

[280] Alsohaimi I.H., Wabaidur S.M., Kumar M., Khan M.A., Alothman Z.A., Abdalla M.A. (2015). Synthesis, characterization of PMDA/TMSPEDA hybrid nano-composite and itsapplications as an adsorbent for the removal of bivalent heavy metals ions. Chem. Eng. J..

[281] Ali A., Mannan A., Hussain I., Hussain I., Zi M. (2018). Effective removal of metal ions fromaquous solution by silver and zinc nanoparticles functionalized cellulose: Isotherm, kineticsand statistical supposition of process. Environ. Nanotechnol. Monit. Manag..

[282] Saad A.H.A., Azzam A.M., El-Wakeel S.T., Mostafa B.B., El-latif M.B.A. (2018). Removal oftoxic metal ions from wastewater using ZnO@Chitosan core shell Nanocomposite. Environ. Nanotechnol. Monit. Manag..

[283] Goyal P., Chakraborty S., Misra S.K. (2018). Multifunctional Fe_3_O_4_-ZnO nanocomposites forenvironmental remediation applications. Environ. Nanotechnol. Monit. Manag..

